# Supplementary material for: Near-infrared uncaging or photosensitizing dictated by oxygen tension
Source: Nat Commun. 2016 Nov 17;7:13378. doi: 10.1038/ncomms13378 (PMC5476797; doi:10.1038/ncomms13378)
Supplement: Supplementary Information — Supplementary Figures 1-27, Supplementary Tables 1-3, Supplementary Methods and Supplementary References [file ncomms13378-s1.pdf]

Supplementary Figures

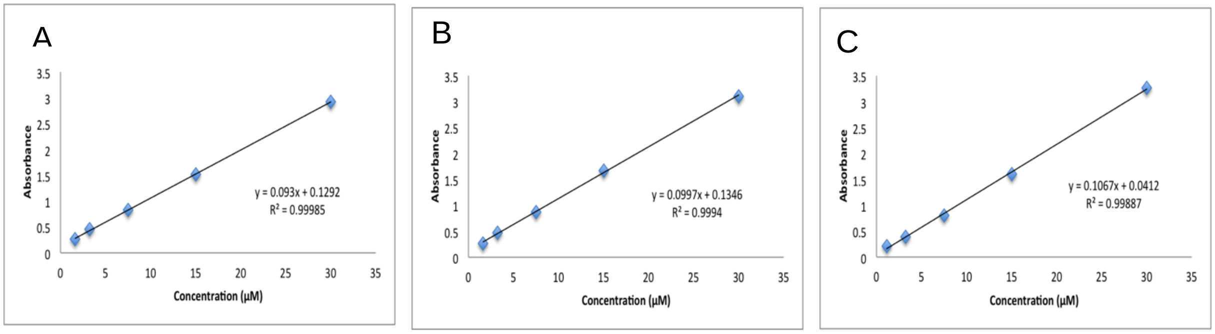

Supplementary Figure 1 - Beer's law plot.
Q-band absorbance at λ_{abs} vs. concentration for **2** (A), **3** (B), and **4** (C) in PBS (50 mM, pH 7.5).

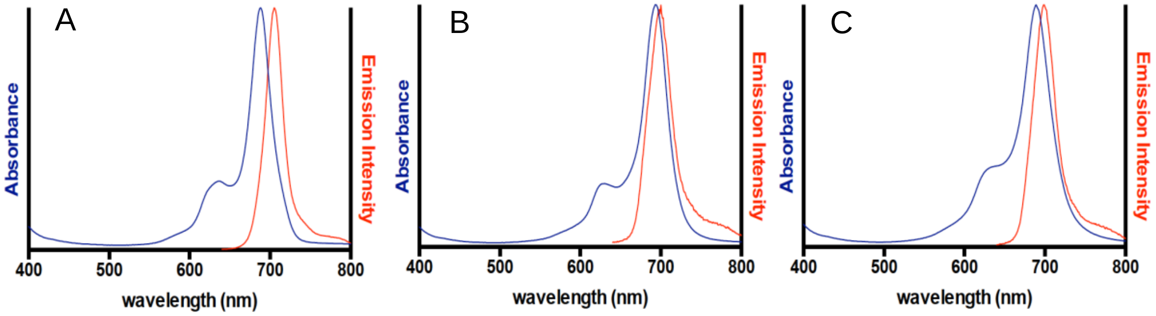

Supplementary Figure 2 – Absorption and fluorescence curves of SiPcs 2-4.
Normalized absorption and emission curves of 5 μM **2** (A), **3** (B), and **4** (C) in PBS (50 mM, pH 7.5). Samples were excited at 615 nm and emission read from 640-800 nm. Normalized curves are shown.

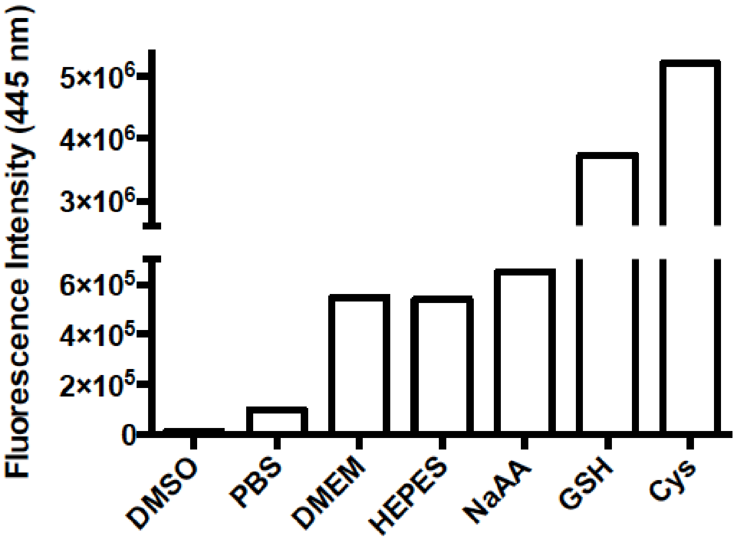

Supplementary Figure 3 - Uncaging as a function of electron donor.
The fluorescence intensity at 445 nm after 10 min of irradiation with 20 mW cm^{-2} 690 nm light is shown. Solutions were as follows: 25 µM **2** in DMSO; 25 µM **2** in 50 mM PBS (pH 7.5); 25 µM **2** in DMEM; 25 µM **2** in 4-(2-hydroxyethyl)-1-piperazineethanesulfonic acid (HEPES) (50 mM, pH 7.5); 25 µM **2** with 5 mM sodium ascorbate (NaAA) in PBS (50 mM, pH 7.5); 25 µM **2** with 5 mM GSH in PBS (50 mM, pH 7.5); 25 µM **2** with 5 mM Cys in PBS (50 mM, pH 7.5). All solutions were purged for 15 minutes with Ar prior to irradiation. Donor reduction potentials: NaAA = 0.11[1], GSH = -0.50[2], Cys = -0.47[3]. Values reported vs NHE were converted to SCE using V(NHE) - 244 mV = V (SCE).

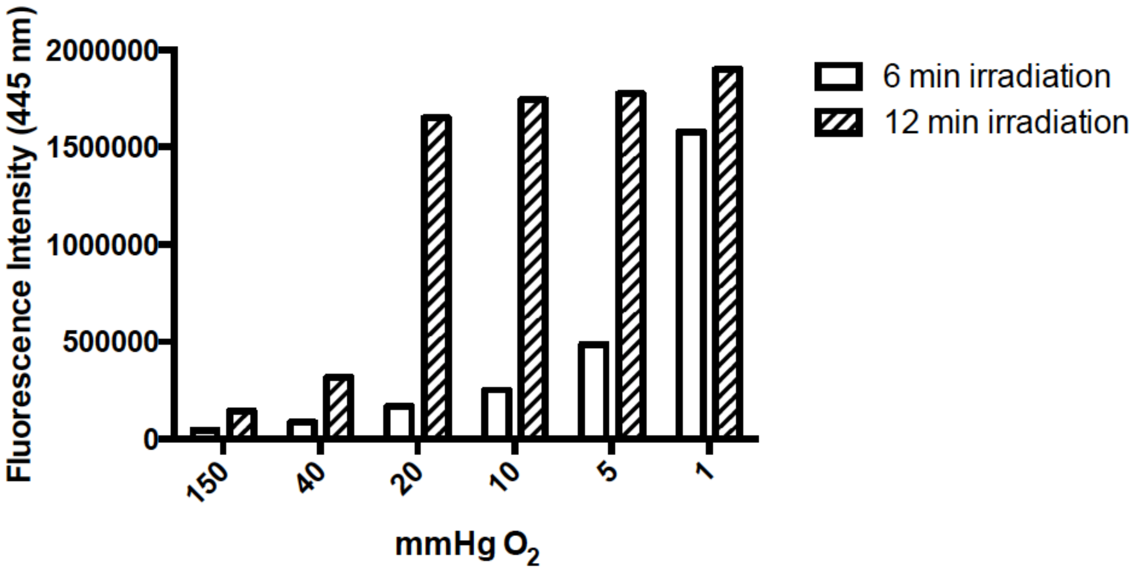

Supplementary Figure 4 - Uncaging as a function of initial solution oxygen concentration.

The fluorescence intensity at 445 nm after 6 or 12 min of irradiation with 25 mW cm^{-2} 690 nm light is shown. A 19 mL solution of **2** (10 µM) in PBS (pH 7.5, 50 mM) with GSH (5 mM) was added to a 20 mL scintillation vial fitted with a contactless oxygen sensor (Pyro Science). Solution oxygen levels were monitored using a fiber optic oxygen meter. Oxygen levels were adjusted by bubbling Ar through the solution (balloon). After oxygen saturation had reached the desired level, the solution was irradiated at 25 mW cm^{-2} with a 690 nm LED for 6 min or 12 min. After irradiation, the umbelliferone fluorescence was read (360 nm excitation/445 nm emission) on a fluorometer.

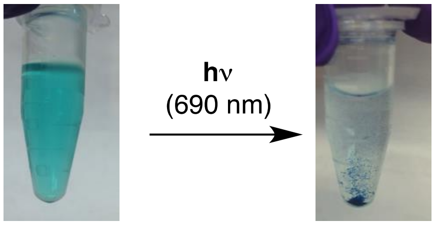

Supplementary Figure 5 - SiPc photoproduct.

SiPc **2** (25 µM in 50 mM pH 7.5 PBS, 5 mM GSH) before (left) and after (right) 20 min of 20 mW cm^{-2} 690 nm irradiation.

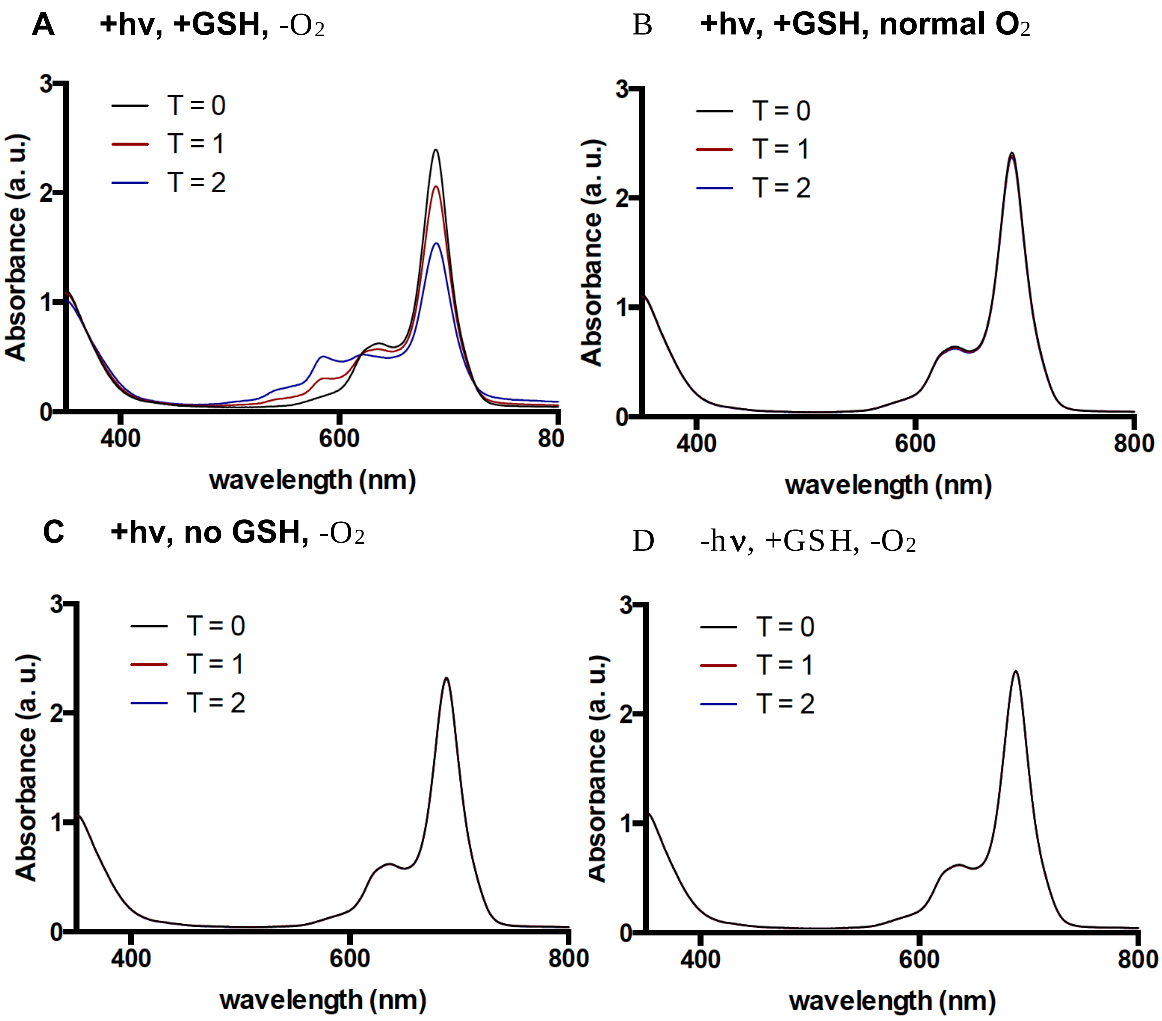

Supplementary Figure 6 - Conditional radical anion formation.

Solutions of **2** (25 µM in 50 mM pH 7.5 PBS, ± 5 mM GSH) were prepared. In noted cases ($-O_2$), samples were deoxygenated by bubbling Ar (balloon) through the septum cap of the sealed cuvette for 20 min prior to irradiation. Where indicated (+hν), samples were irradiated with 20 mW cm^{-2} 690 nm light for 1 min, measured, then submitted to a second 1 min of light and measured. (A) +hν, 5 mM GSH, $-O_2$ (B) +hν, 5 mM GSH, no deoxygenation (C) +hν, no GSH, $-O_2$ (D) -hν, 5 mM GSH, $-O_2$.

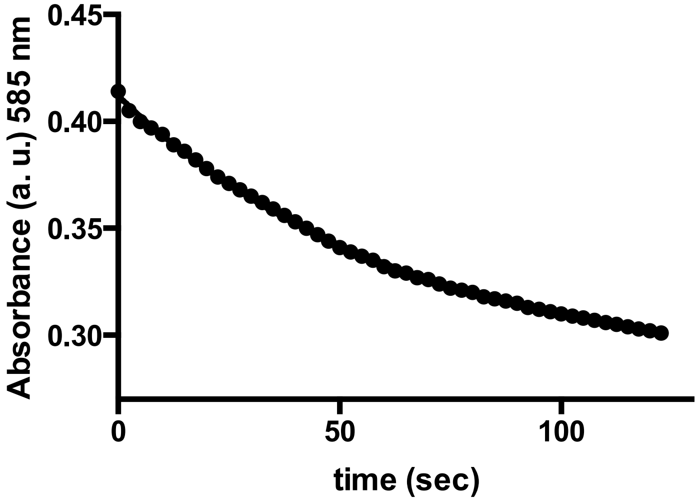

Supplementary Figure 7 - Radical anion decay.
A solution of **2** (25 μM in 50 mM pH 7.5 PBS with 5 mM GSH) was deoxygenated by bubbling Ar (balloon) through the septum cap of a sealed quartz cuvette for 20 min, followed by 690 nm irradiation at 20 mW cm^{-2} for 90 s. After irradiation, the absorbance at 585 nm was monitored on a spectrophotometer.

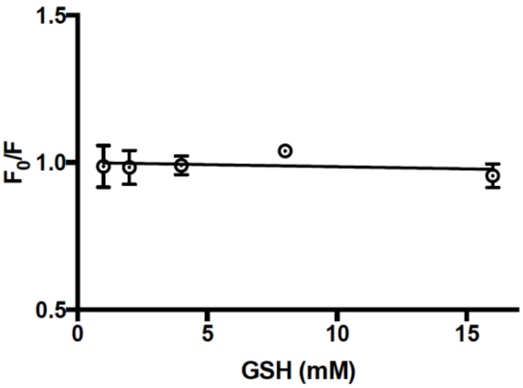

Supplementary Figure 8 - Stern-Volmer analysis.
Solutions of SiPc **2** (1 μM in 50 mM pH 7.5 PBS) with varying amounts of GSH (1, 2, 4, 8, and 16 mM) were prepared and the SiPc fluorescence was read on a fluorimeter (ex. 615 nm/em. 650-740 nm). The integrated area of the fluorescence spectra from 650-740 nm of a solution of **2** with no added GSH (F_0) was divided by that of each sample solution (F) and plotted against the GSH concentration.

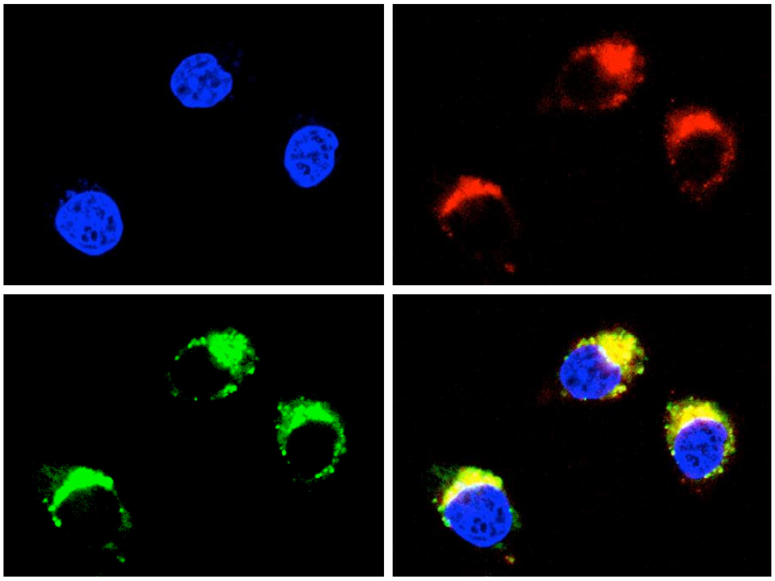

Supplementary Figure 9 - Confocal microscopy images.
Clockwise from top left: fluorescence emission from Hoechst, fluorescence emission from **3**, fluorescence emission from LysoTracker, and overlay. HeLa cells (5 x 10^4) were plated on Nunc Lab-Tek II chambered #1.5 German borosilicate coverglass (Thermo Fisher Scientific, Inc.) and allowed to adhere overnight. Cells were incubated with 200 nM **3** for 3 h, washed twice with PBS, incubated with 1 μM Hoechst 33342 for 0.5 h, washed twice with PBS, incubated with 500 nM LysoTracker Green DND-26 (Life Technologies) for 0.5 h, washed twice with PBS, and imaged. Fluorescence microscopy was performed using a Zeiss LSM 780 confocal microscope at 63x magnification using a plan-apochromat oil immersion objective. **3** was imaged using a HeNe633 laser (633 nm excitation, 650 nm longpass emission), LysoTracker using an Argon/2 laser (488 nm excitation, 505-550 bandpass emission), and Hoechst using a Diode 405-30 laser (405 nm excitation, 420-480 nm bandpass emission). Image processing was conducted with Fiji.

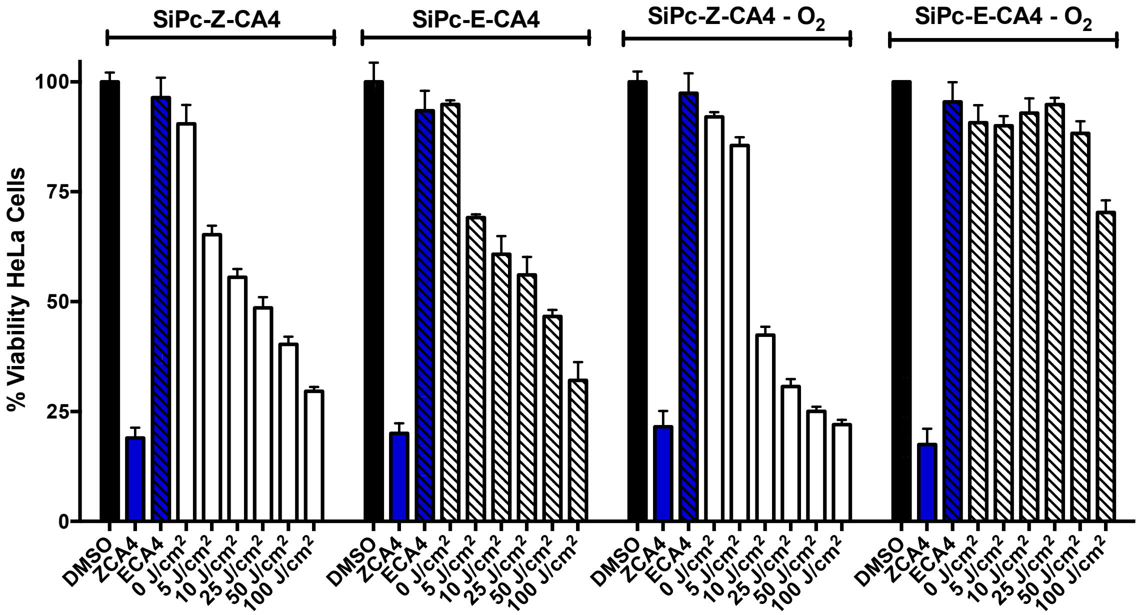

Supplementary Figure 10 - Effect of light dose on cell viability with 3 and 4 (± O_2).
Cells in the presence of DMSO, **3**, **4**, *Z*-CA4, and *E*-CA4 were exposed to 0, 5 (4 min), 10 (8 min), 25 (21 min), and 50 J cm^{-2} (42 min) of 20 mW cm^{-2} 690 nm light under normoxic (ambient air) and hypoxic (N_2 stream through a sealed modular incubator chamber) conditions. Experiments were conducted in quadruplicate, with error bars representing the standard deviation. All other assay parameters and viability analysis are as described in the Methods section.

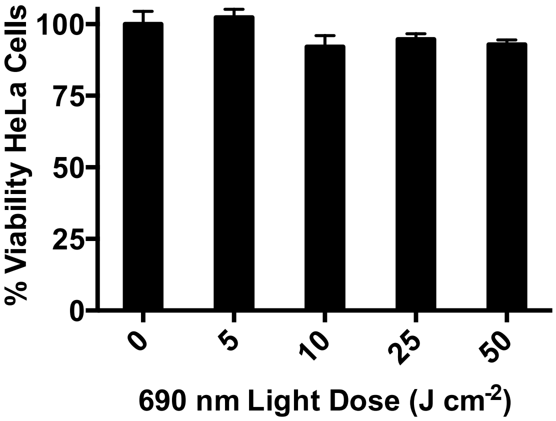

Supplementary Figure 11 - Effect of irradiation on cell viability.
Cells were exposed to the indicated doses of 20 mW cm^{-2} 690 nm light by varying the irradiation time. Cells in the absence of inhibitor (e.g. media alone) were exposed to 0, 5 (4 min), 10 (8 min), 25 (21 min), and 50 J cm^{-2} (42 min) of 20 mW cm^{-2} 690 nm light under normoxic conditions (ambient air). Experiments were conducted in quadruplicate, with error bars representing the standard deviation. All other assay parameters and viability analysis are as described in the Methods section.

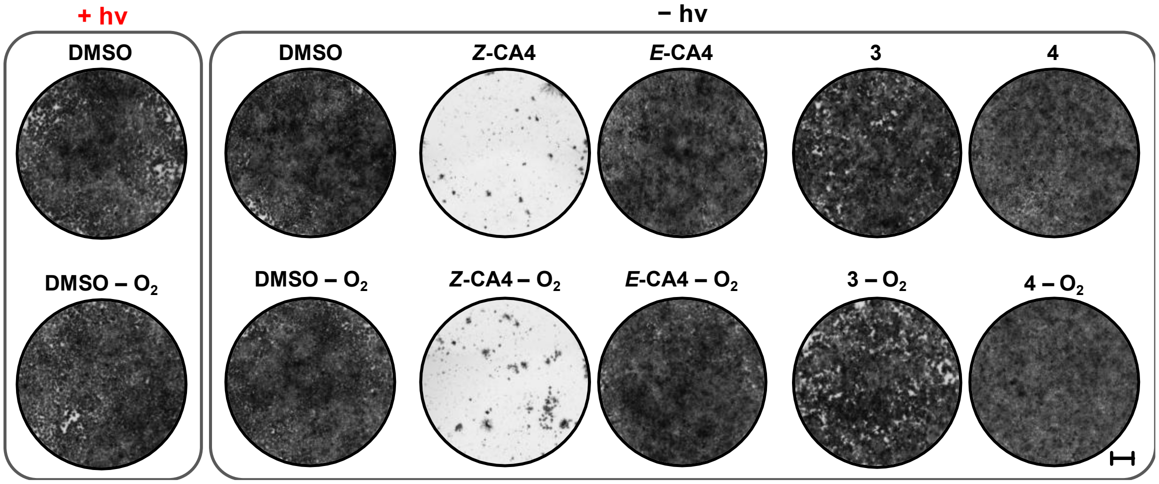

Supplementary Figure 12 - Photopatterning controls.
Cells were treated with 200 nM of the indicated species under normoxic and hypoxic conditions. In the left panel, wells were irradiated in half-well fashion (see Methods and Figure 5a). In the right panel, wells were not irradiated. Cell viability analysis was performed using MTT and wells were imaged under brightfield at 4x magnification. Scale bar = 5 mm. Experiments were conducted in quadruplicate with representative images shown. All other assay parameters and viability and photopatterning analyses are as described in the Methods section.

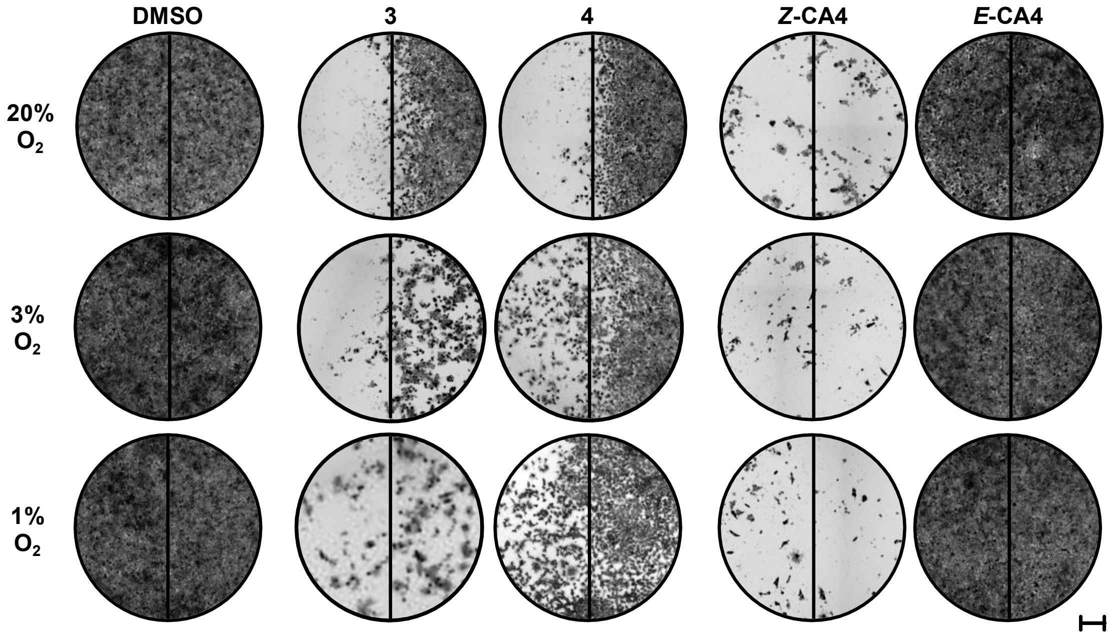

Supplementary Figure 13 - Effect of O_2 concentration on photopatterning with 3 and 4.

Cells in the presence of DMSO, **3**, **4**, *Z*-CA4, and *E*-CA4 (200 nM) were exposed to 50 J cm^{-2} (42 min) of 20 mW cm^{-2} 690 nm light in an atmosphere of 1, 3, and 20% O_2. These conditions were achieved by equilibrating plates for 1 h in a humidified 37 ^{o}C incubator set to the relevant % O_2 and 5% CO_2 (balance N_2) prior to irradiation. Cells were irradiated in half-well fashion, treated with MTT, and imaged under brightfield at 4x magnification. Scale bar = 5 mm. Experiments were conducted in quadruplicate with representative images shown. All other assay parameters and viability and photopatterning analyses are as described in the Methods section.

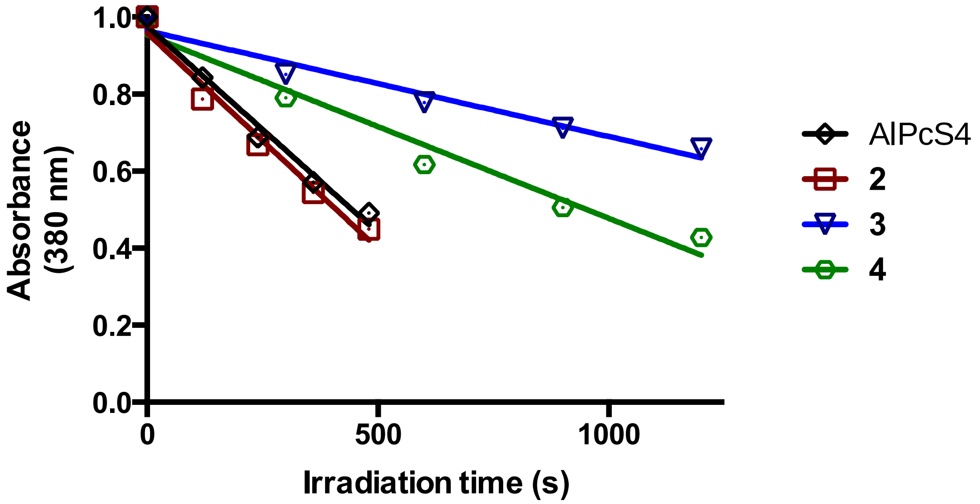

Supplementary Figure 14 - Singlet oxygen quantum yields.

Singlet oxygen ($^1O_2$) quantum yields were evaluated using the relative method with ADMA (9,10-Anthracenediyl-bis(methylene)dimalonic acid)[4] as the $^1O_2$ scavenger and aluminum (III) phthalocyanine chloride tetrasulfonic acid (AlPcS4, $\Phi_\Delta = 0.34$)[5,6] as the standard. A solution of **2, 3, 4**, or AlPcS4 (1 µM) and ADMA (10 µM) in PBS (pH 7.5) was irradiated with a 690 nm LED at 20 mW cm^{-2} in a quartz cuvette. The decrease in ADMA absorption at 380 nm was monitored by UV-Vis spectroscopy. The singlet oxygen quantum yield (Φ_Δ) was calculated using supplementary equation (**1**)[7]

$$\phi_\Delta = \phi_\Delta^{std} \frac{R I_{abs}^{std}}{R^{std} I_{abs}} \qquad (1)$$

where Φ_Δ^{std} is the singlet oxygen quantum yield for the standard, R and R^{std} are the rates of ADMA consumption in the presence of **2**, **3**, or **4** and the standard, AlPcS4, respectively, and I_{abs} and I_{abs}^{std} are the rates of light absorption of **2**, **3**, or **4**, and AlPcS4, respectively. R and R^{std} are determined from the slope of a plot of the normalized absorbance at 380 nm vs. the irradiation time (s). I_{abs} and I_{abs}^{std} are determined from the overlap integral of the radiation light source intensity and the absorbance of the photosensitizer Q-band.[7]

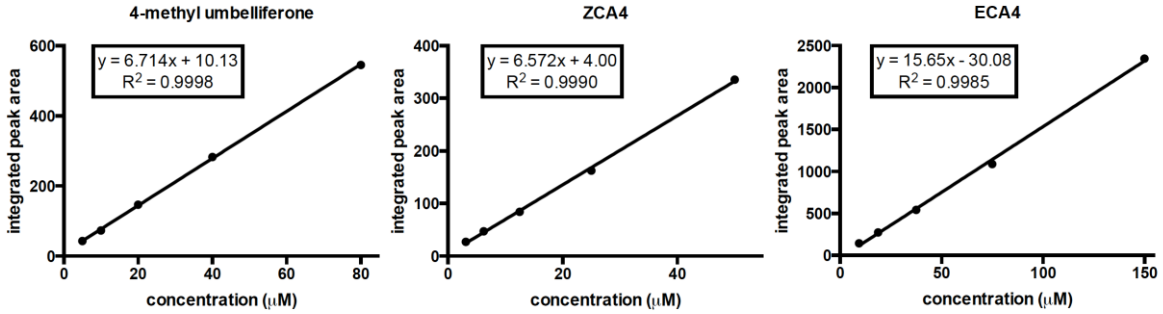

Supplementary Figure 15 – HPLC analysis of photouncaging yields
The yield of ligand release was quantified using an external calibration HPLC method. Calibration curves were made for each ligand: 4-methylumbelliferone, Z-CA4 and *E*-CA4 (above). Samples of **2**, **3**, or **4** (25 μM in DMEM ± 5 mM GSH) were irradiated in a quartz cuvette with a septum screw cap using a 690 nm LED at 20 mW cm^{-2}. All experiments were run in triplicate. In noted cases (-O_2), samples were deoxygenated by bubbling Ar (balloon) through the septum cap of the sealed cuvette for 20 min prior to irradiation. Unirradiated control experiments were run in parallel. The internal temperature of the samples did not exceed 25 °C over the irradiation time course. Following irradiation (or dark incubation time), 250 μL of the sample solution was diluted twofold (250 μL H_2O in the case of **2** and 250 μL MeCN for **3** or **4**), vortexed, transferred to a LCMS vial, and analyzed by HPLC as shown with representative traces below.

Supplementary Figure 16 – HPLC Data for 2

A. Trace of 4-methylumbelliferone

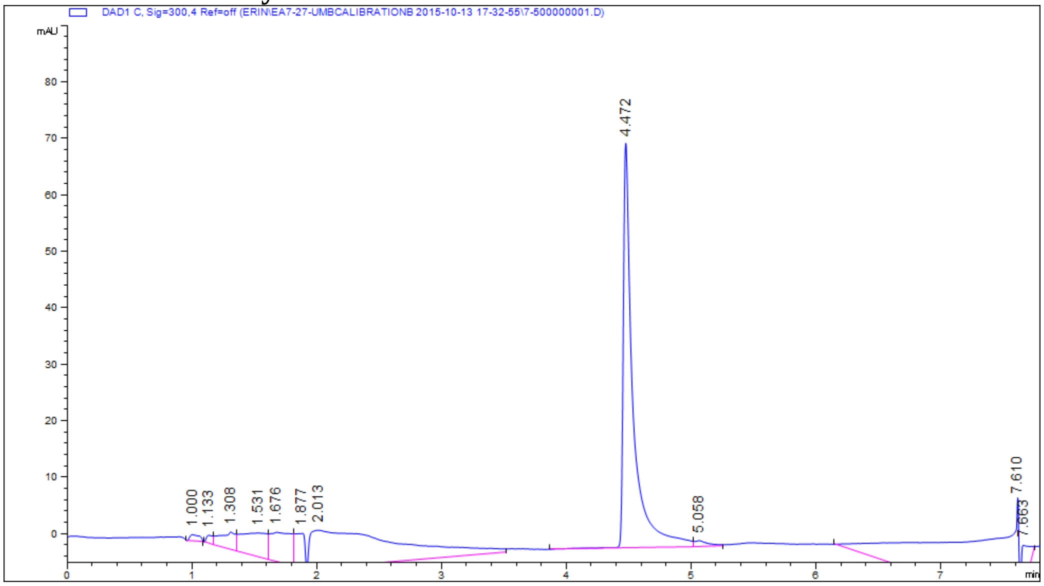

B. Trace of photolysis at T = 10 min

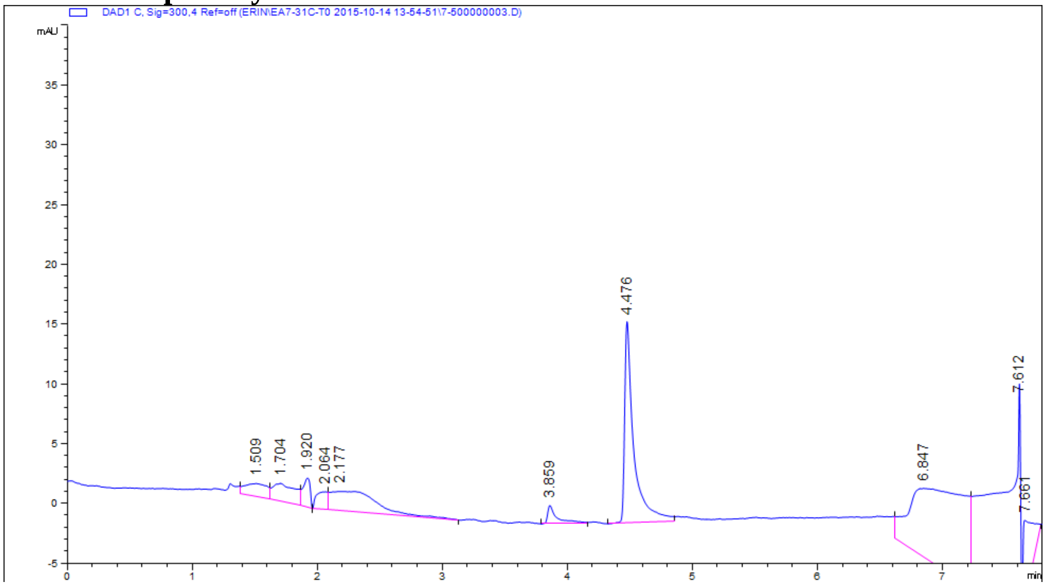

Supplementary Figure 17 – HPLC Data for 3

A. Trace of *Z*-CA4

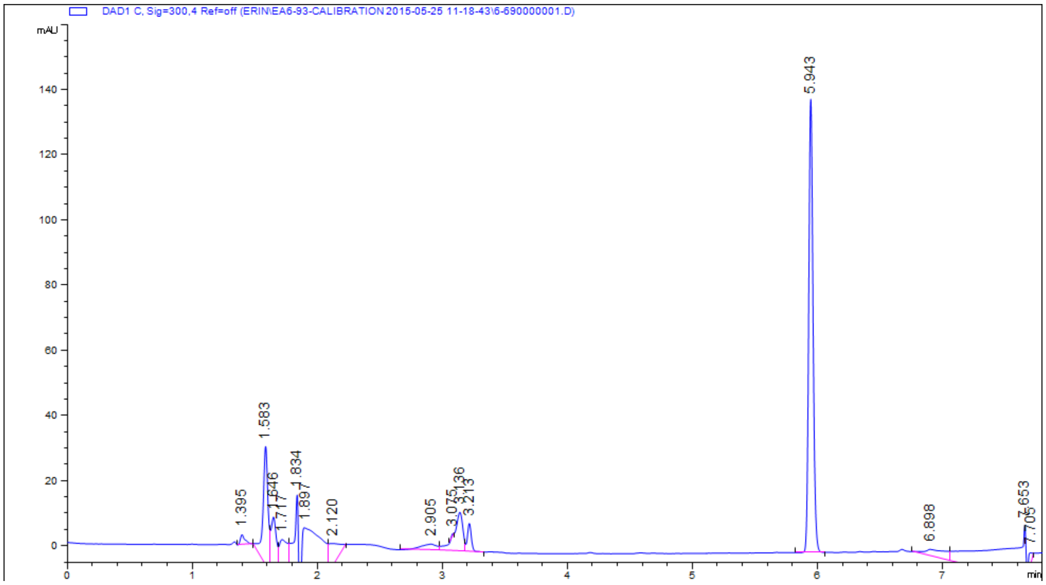

B. Trace of photolysis at T = 30 min

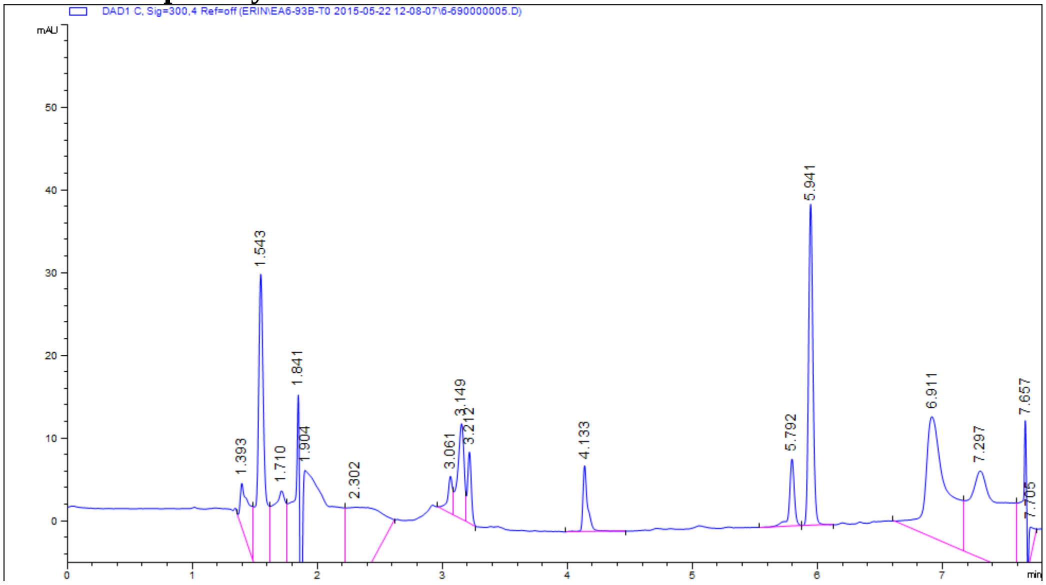

Supplementary Figure 18 – HPLC Data for 4

A. Trace of *E*-CA4

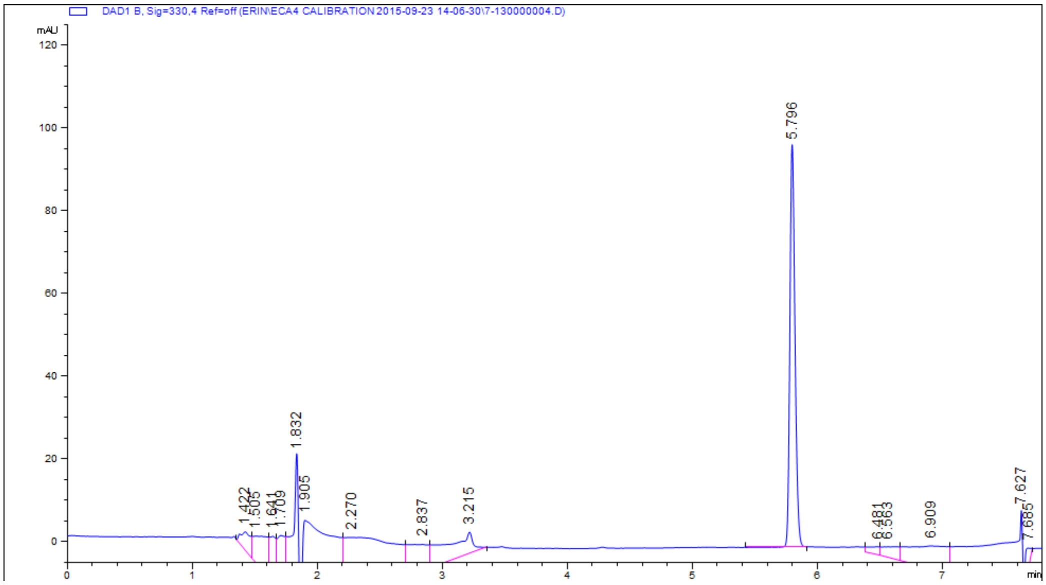

B. Trace of Photolysis at T = 30 min

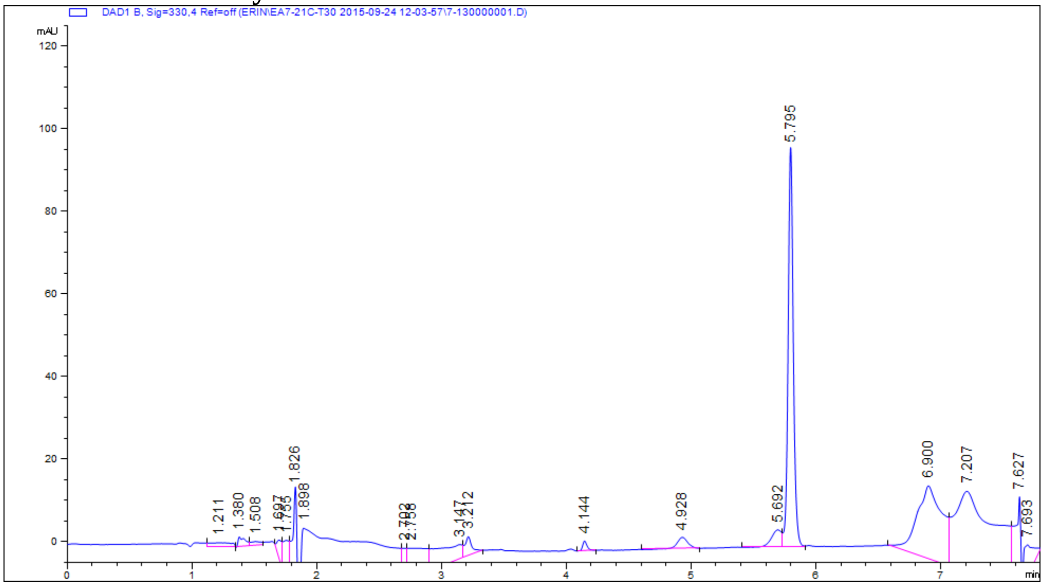

Supplementary Figure 19 - NMR spectra for SiPc[$C_3H_5(NMe_2)_2O$]($C_{10}H_7O_3$).

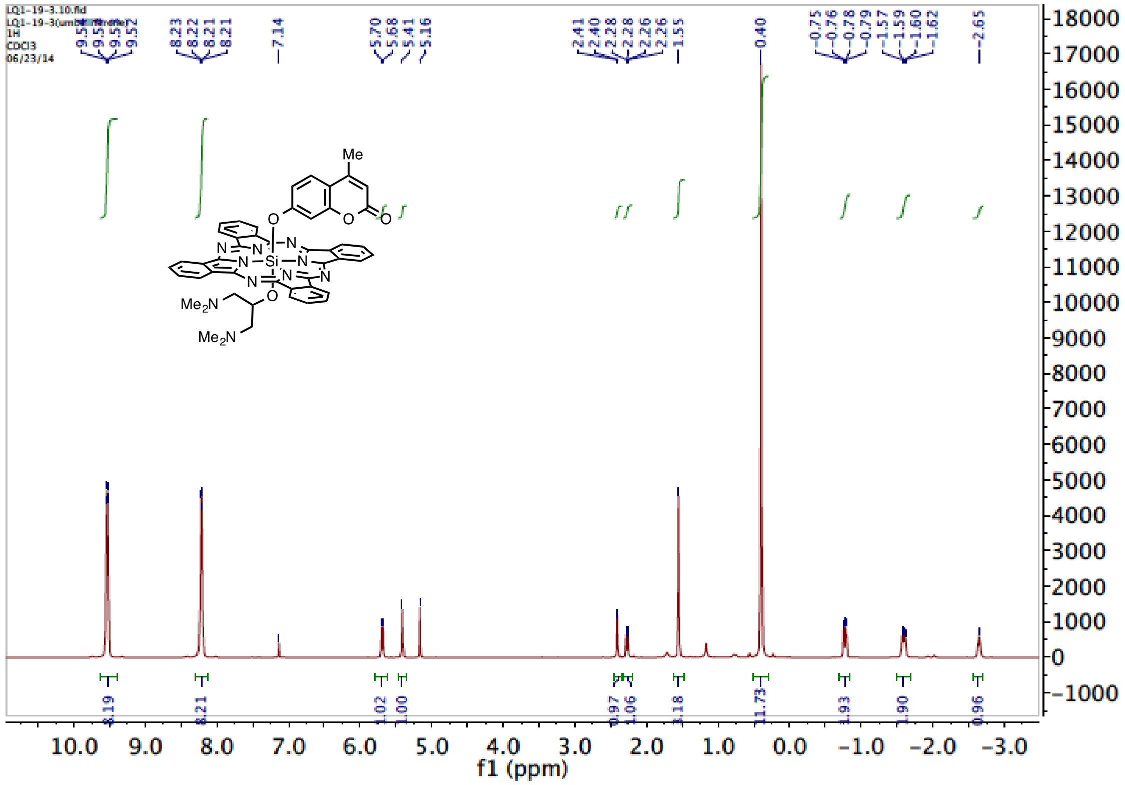

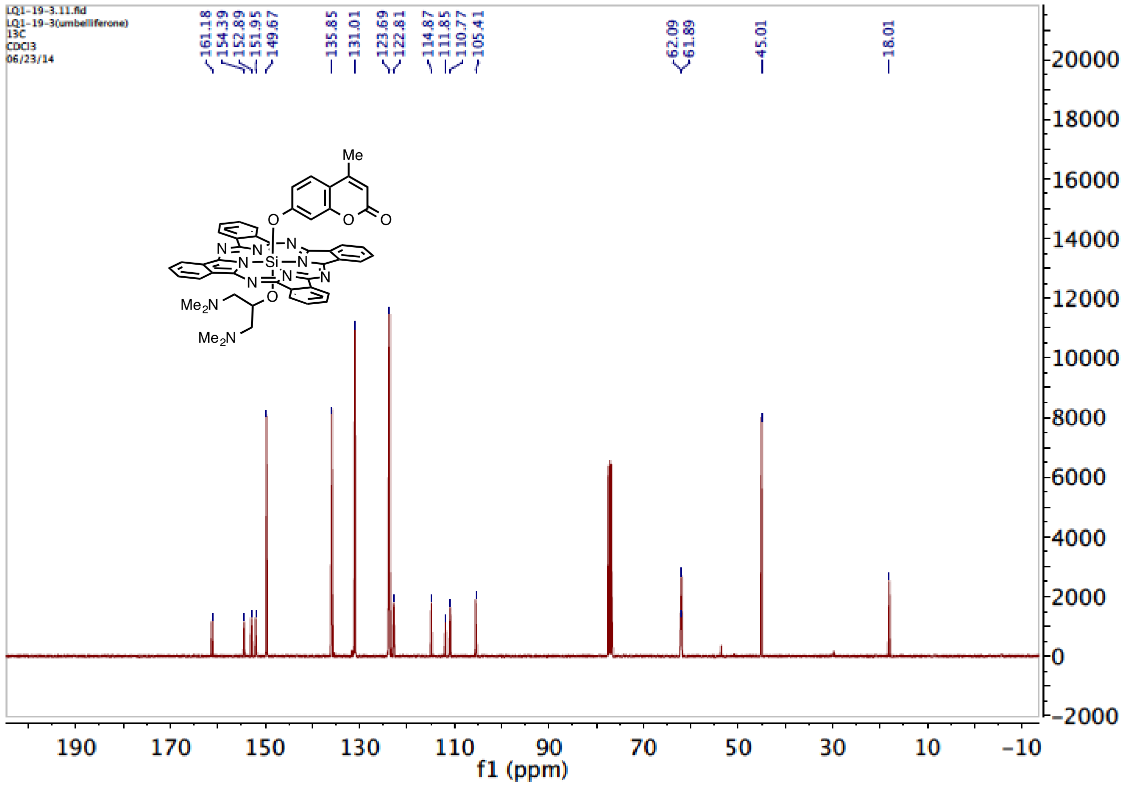

Supplementary Figure 20 - NMR spectra for {SiPc[$C_3H_5(NMe_3)_2O$]($C_{10}H_7O_3$)}I_2 (2).

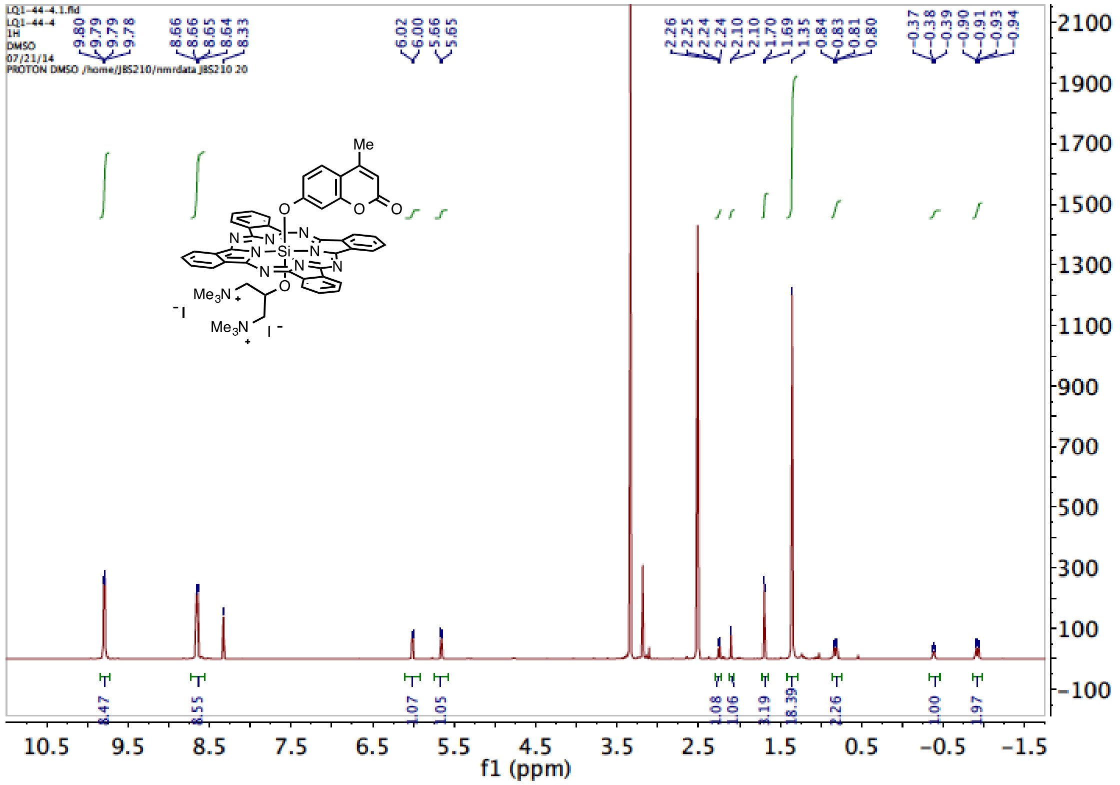

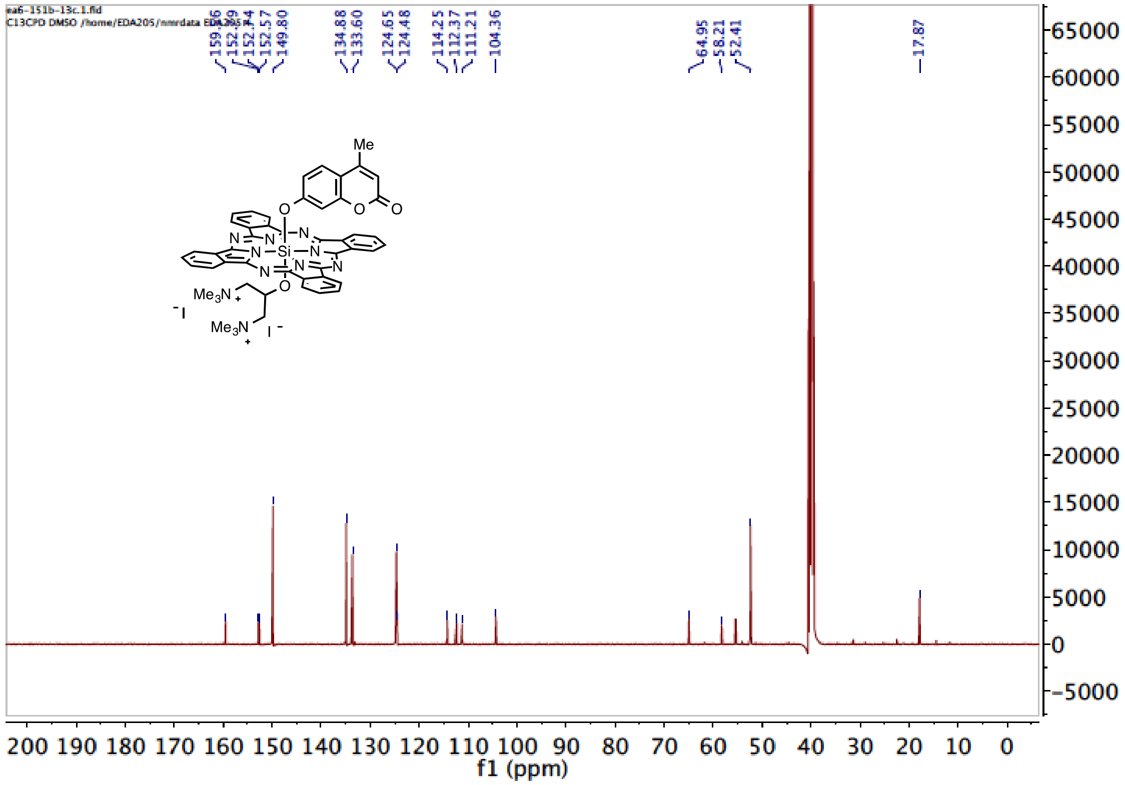

Supplementary Figure 21 - NMR spectra for SiPc[$C_3H_5(NMe_2)_2O$]($C_{18}H_{19}O_5$).

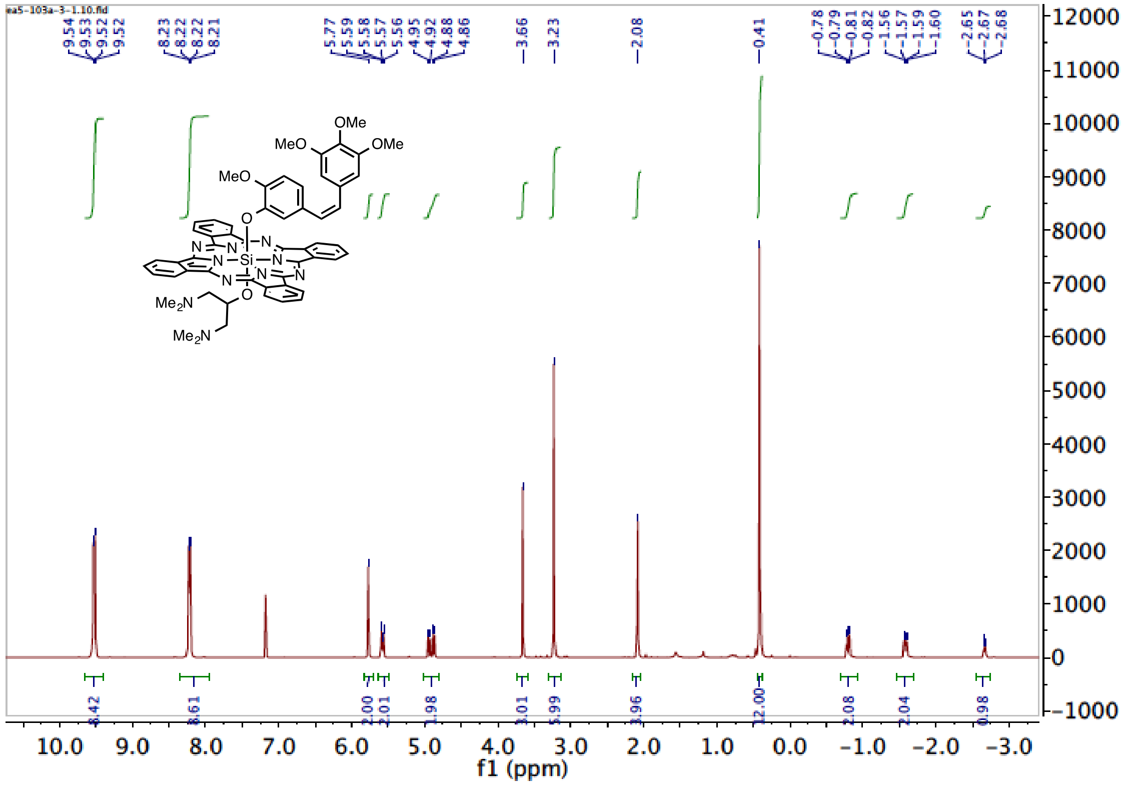

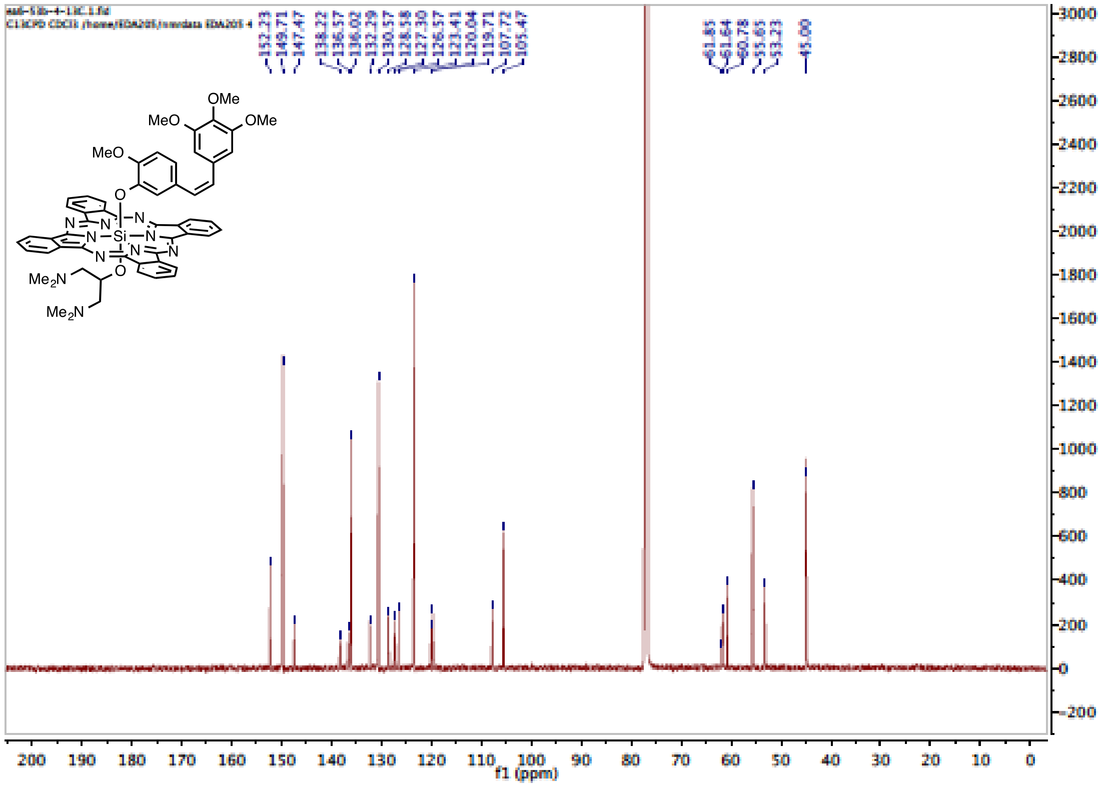

Supplementary Figure 22 - NMR spectra for $\{SiPc[C_3H_5(NMe_3)_2O](C_{18}H_{19}O_5)\}I_2$ (3).

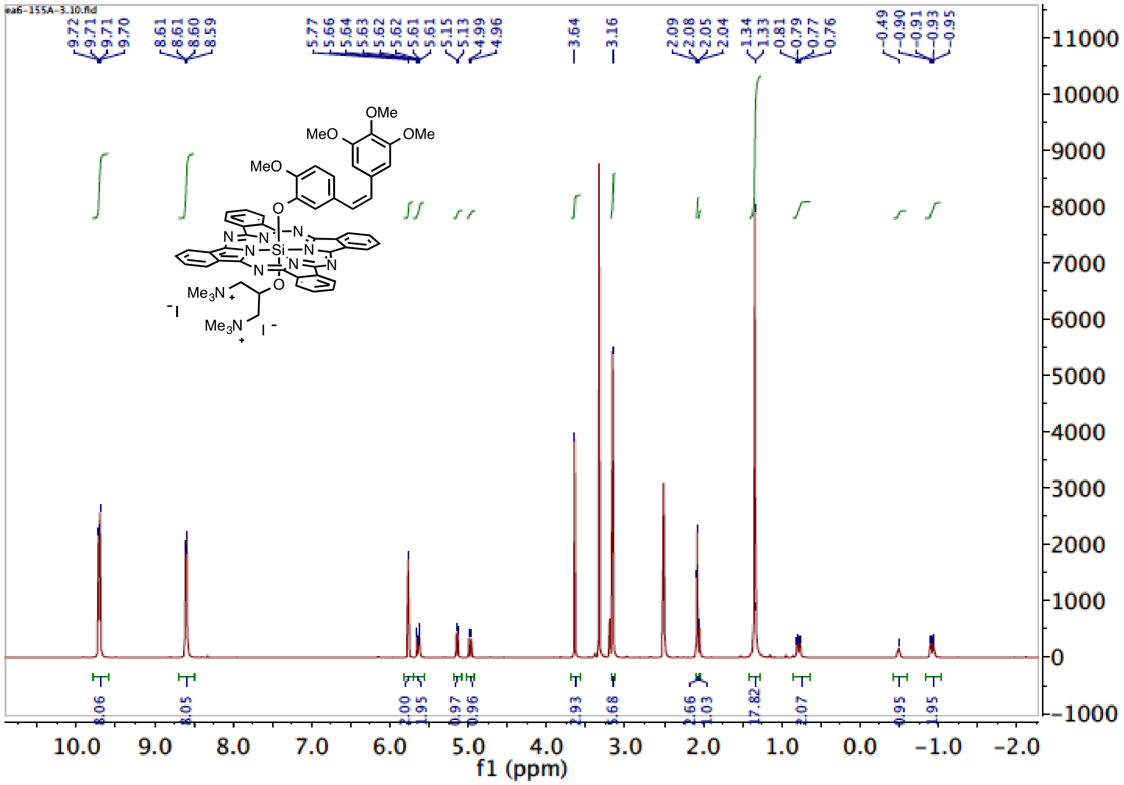

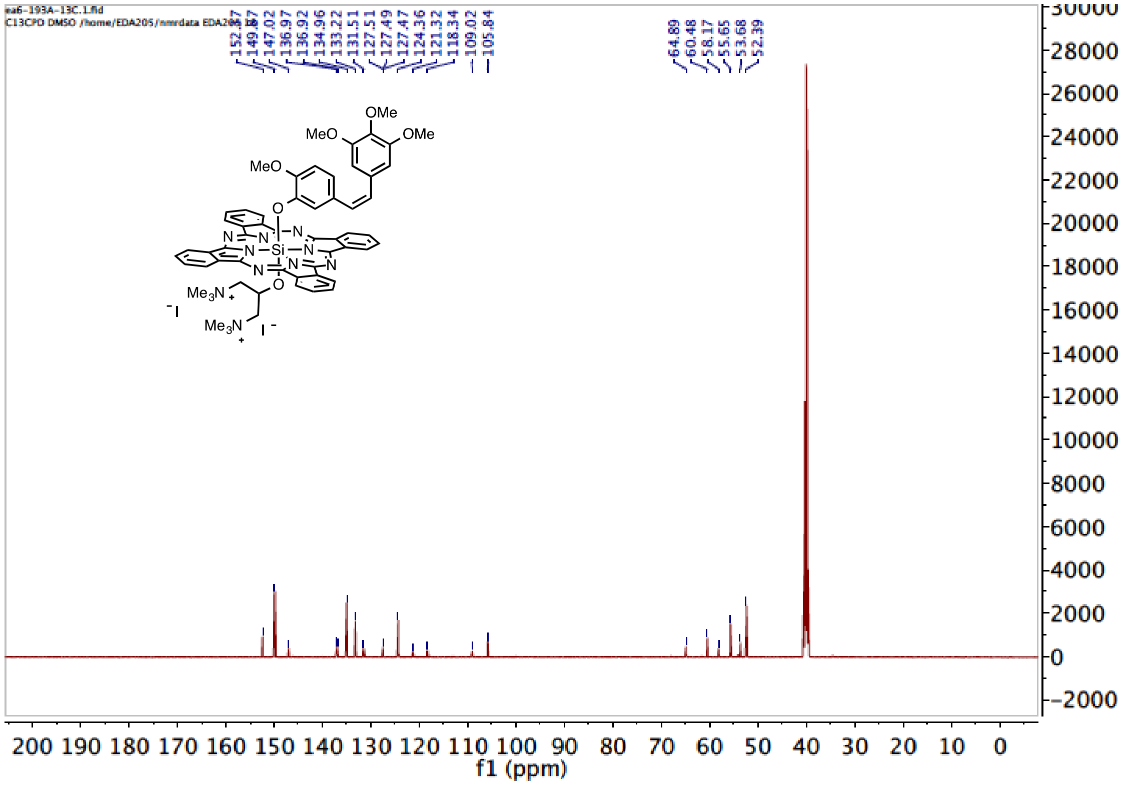

Supplementary Figure 23 - NMR spectra for SiPc[$C_3H_5(NMe_2)_2O$](E-CA4).

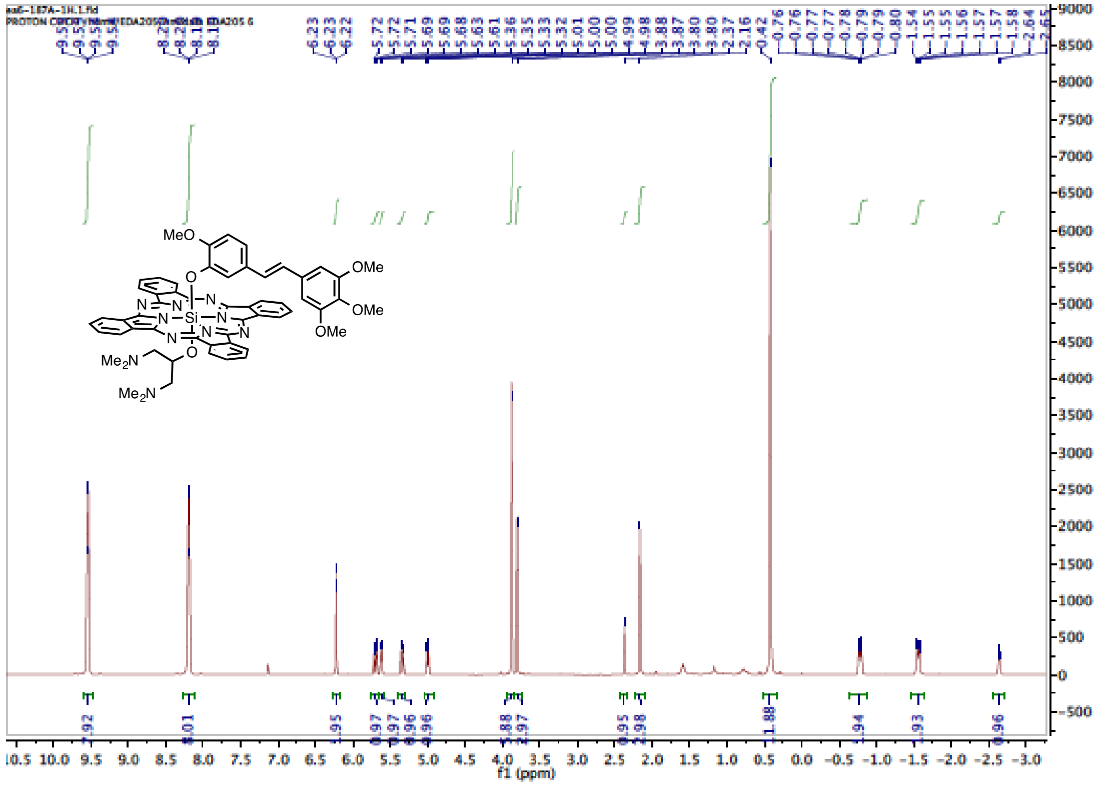

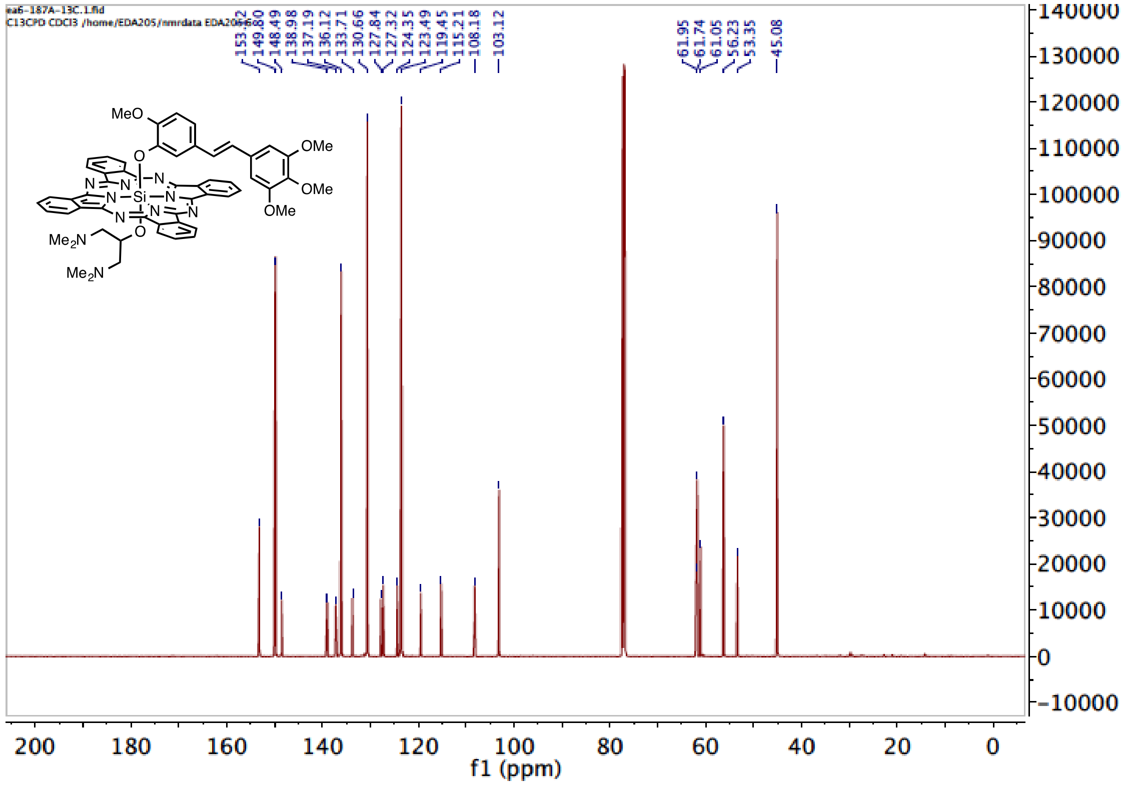

Supplementary Figure 24 - NMR spectra for {SiPc[$C_3H_5(NMe_3)_2O$](E-CA4)}I_2 (4).

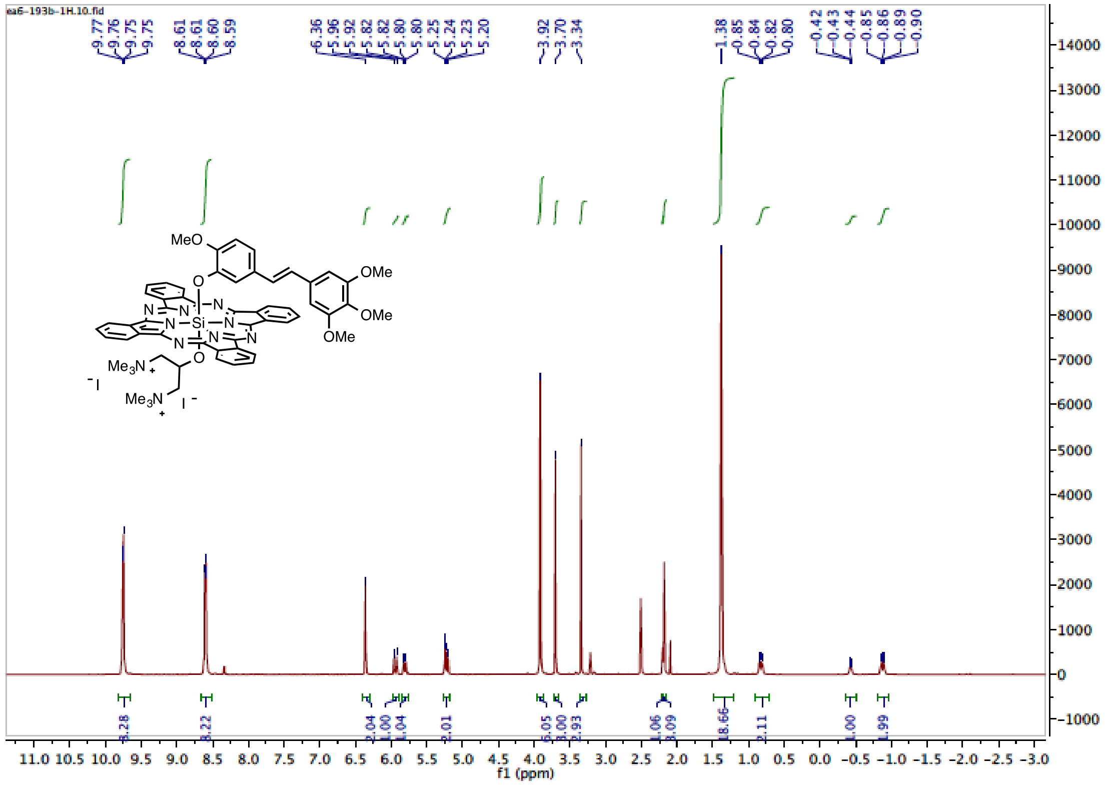

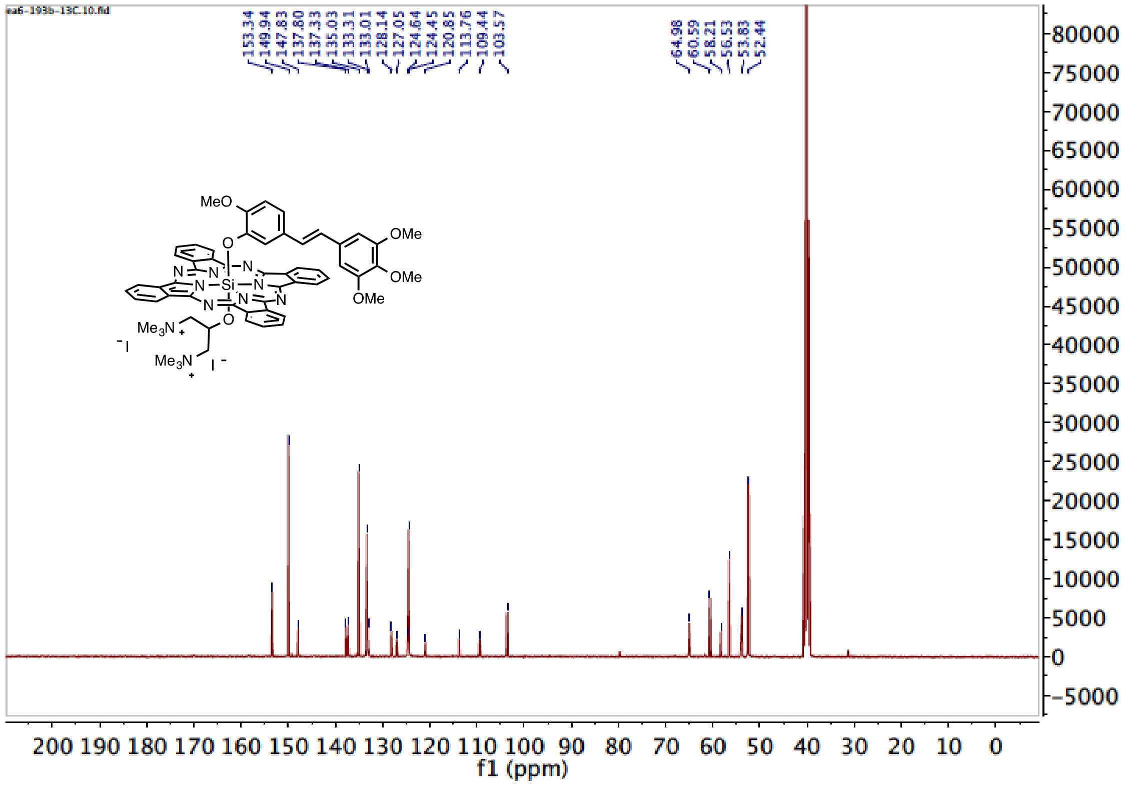

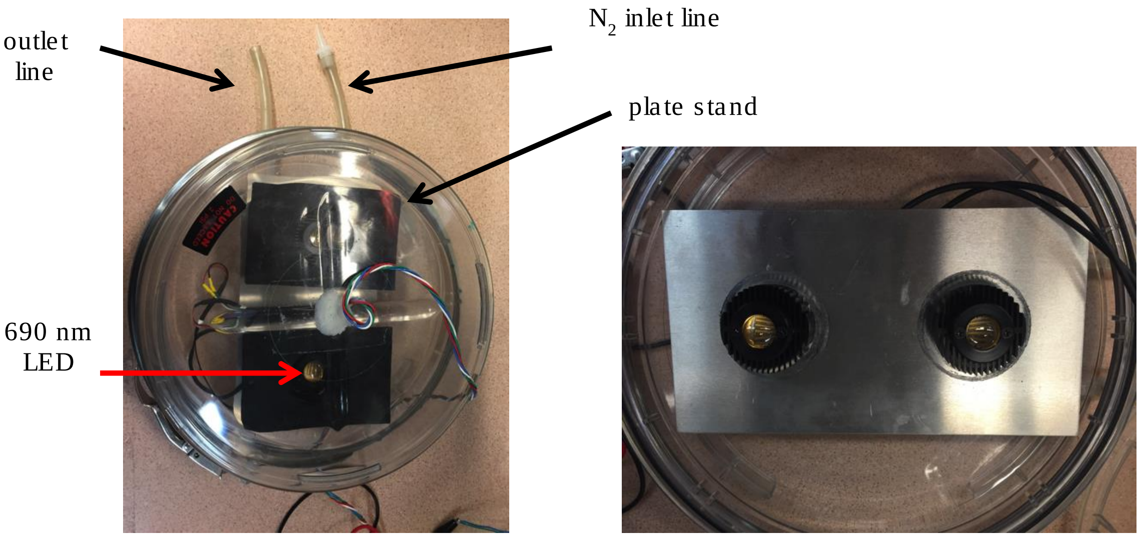

Supplementary Figure 25 - Modular incubator chamber fitted with 2 690 nm LEDs.

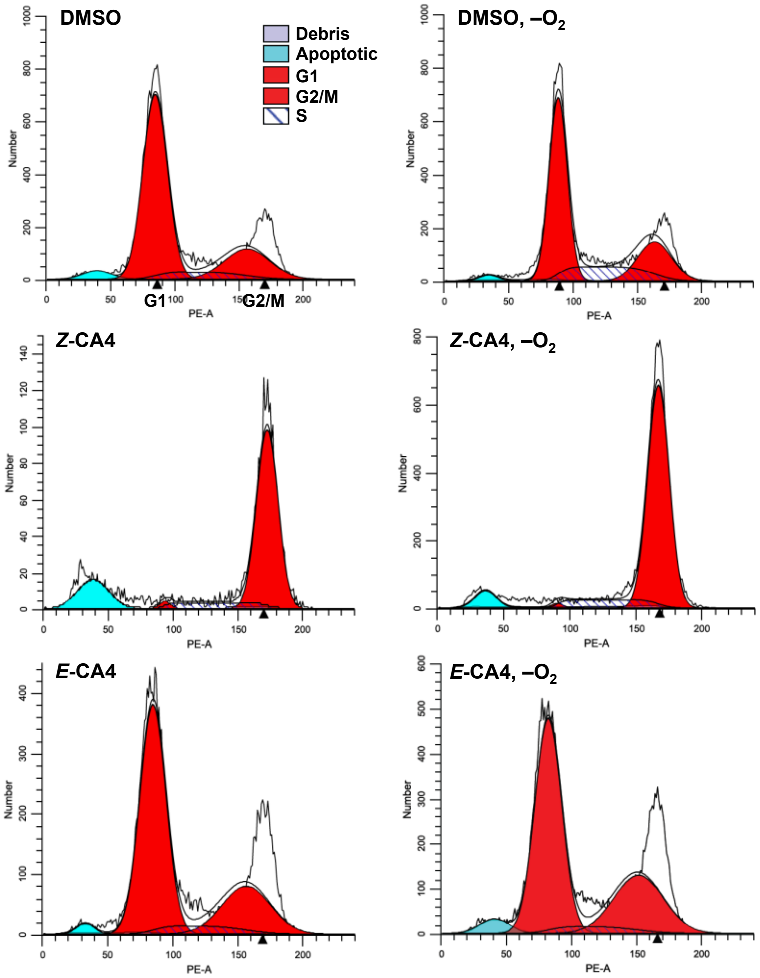

Supplementary Figure 26 - Deconvoluted histograms showing cell cycle analysis of HeLa cells treated with 200 nM *Z*-CA4, *E*-CA4, or DMSO in the absence of irradiation.

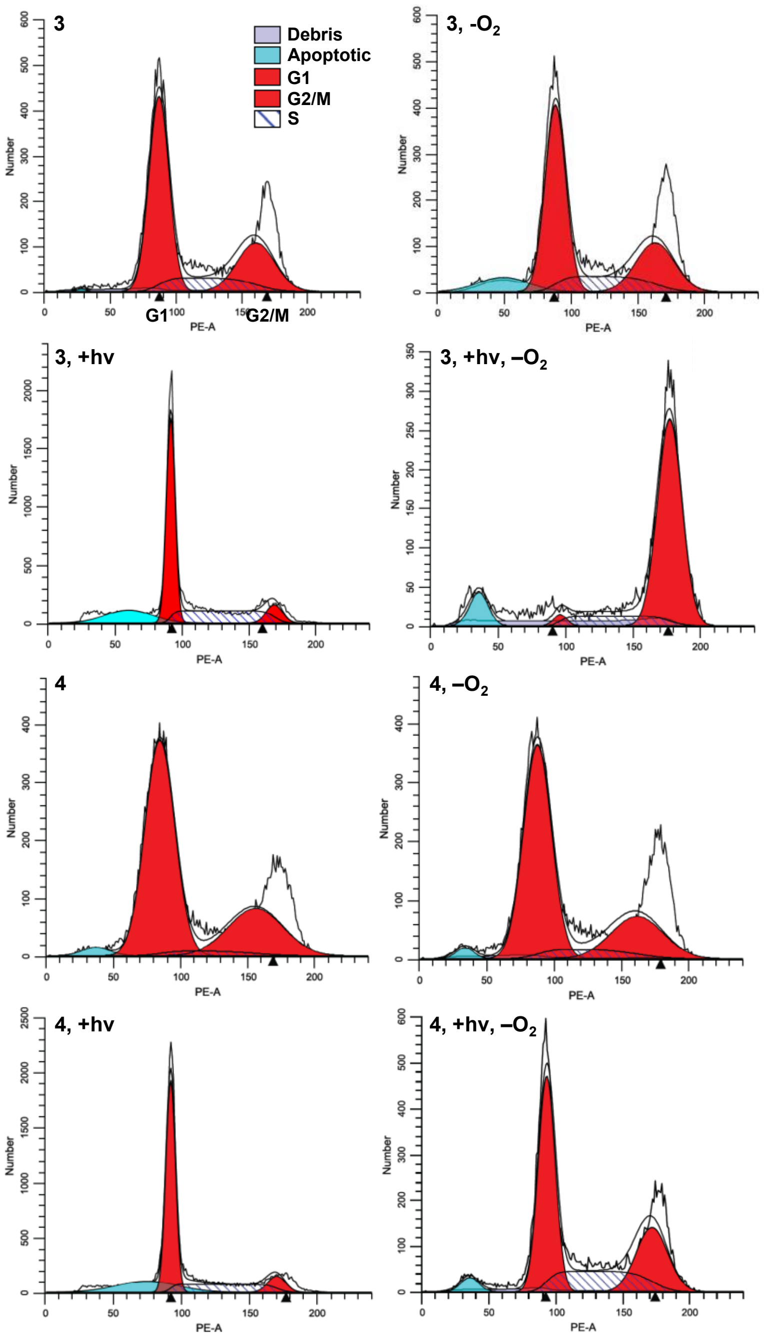

Supplementary Figure 27 - Deconvoluted histograms showing cell cycle analysis of HeLa cells treated with 200 nM 3 or 4 in the presence and absence of irradiation and under normoxic and hypoxic conditions.

Supplementary Tables

Supplementary Table 1 - Photodegradation quantum yields.

The role of experimental setup in obtaining Φ_{PD} values was pointed out in a recent paper.[8] We evaluated two known photosensitizers with similar λ_{max}'s with reported values for Φ_{PD} (verteporfin and IRDye 700DX).[9,10] In our studies we have used a low power LED light source, which is quite different than the 100 mW and 1 W lasers used in the other studies. Likely as a consequence in both cases, the estimates we obtained fall only within approximately an order of magnitude of the reported values.

Compound (Conditions)	Φ_{PD}
**2** (20 µM in 50 mM PBS, pH 7.5, 5 mM GSH, deoxygenated)	2.7×10^{-3}
**2** (20 µM in 50 mM PBS, pH 7.5)	2.9×10^{-6}
**Verteporfin** (20 µM in 50 mM PBS, pH 7.5)	5.0×10^{-6}
**Verteporfin** from ref. 9	2.8×10^{-5}
**IRDye 700DX** (20 µM in 50 mM PBS, pH 7.5)	1.0×10^{-6}
**IRDye 700DX** from ref. 10	1.6×10^{-7}

Supplementary Table 2 - O_2 quench of radical anion.

Solutions of **2** (25 µM in 50 mM pH 7.5 PBS with 5 mM GSH) were deoxygenated by bubbling Ar (balloon) through the septum cap of a sealed quartz cuvette for 20 min. The absorbance of the SiPc Q-band (A_{690}) in each sample was recorded on a spectrophotometer. The sample was irradiated with 690 nm light (20 mW cm^{-2}) for the time indicated below (left column) and the Q-band absorbance was recorded again. O_2 (balloon) was then bubbled through the sample for 1 min and a final Q-band absorbance reading obtained. Measurements were performed in triplicate.

**Irradiation Time (s)**	**%A_{690}^0 after hν**	**%A_{690}^0 after hν and 1 min +O_2**
60	88.7 ± 1%	95.0 ± 0.5%
120	68.1 ± 3%	85.7 ± 3%
180	50.7 ± 6%	67.8 ± 6%

Supplementary Table 3 - Cell cycle analysis.

Cell cycle-specific DNA content of HeLa cells treated with 200 nM **3**, **4**, *Z*-CA4, and *E*-CA4 in the presence and absence of irradiation and under normoxic and hypoxic conditions.

			DNA Content					
			**sub-G1 (Apoptotic)**	**G1**	**S**	**G2/M**	**% CV**	**Predominant Stage**
**DMSO**	**− hv**	**+ O_2**	3.7%	70.7%	8.0%	21.3%	11.3%	G1
		**− O_2**	1.8%	58.3%	18.7%	23.0%	8.0%	G1
***Z*-CA4**	**− hv**	**+ O_2**	5.9%	1.9%	11.3%	86.8%	4.8%	G2/M
		**− O_2**	6.1%	0.9%	11.5%	87.6%	4.9%	G2/M
***E*-CA4**	**− hv**	**+ O_2**	2.0%	67.4%	6.7%	25.9%	11.9%	G1
		**− O_2**	4.5%	63.2%	5.3%	31.5%	12.6%	G1
**3**	**− hv**	**+ O_2**	0.3%	58.1%	15.2%	26.7%	8.8%	G1
		**− O_2**	4.3%	55.7%	17.4%	26.9%	8.8%	G1
	**+ hv**	**+ O_2**	16.2%	58.3%	32.3%	9.4%	3.6%	G1*
		**− O_2**	5.1%	2.4%	13.6%	84.0%	5.1%	G2/M
**4**	**− hv**	**+ O_2**	2.4%	68.5%	3.9%	27.6%	13.2%	G1
		**− O_2**	2.1%	67.3%	8.2%	24.5%	11.9%	G1
	**+ hv**	**+ O_2**	18.3%	68.2%	22.5%	9.3%	3.8%	G1*
		**− O_2**	3.3%	49.6%	23.0%	27.4%	6.9%	G1

*indicates elevated level of apoptotic cells

Supplementary Methods

General Materials and Methods. All commercially obtained reagents were used as received, with the exception of chloroform (ethanol-free), which was passed through solid potassium carbonate before use. 9,10-Anthracenediyl-bis(methylene)dimalonic acid (ADMA) was obtained from Sigma-Aldrich. Aluminum (III) phthalocyanine chloride tetrasulfonic acid (AlPcS4) was purchased from Frontier Scientific, Inc. Flash column chromatography was performed using basic alumina (60 Å, 50−200 micron particle size, Redisep® Rf Alumina Basic Columns) on a CombiFlash® Rf 200i (Teledyne Isco, Inc). ^{1}H and ^{13}C NMR spectra were recorded on Bruker spectrometers (at 400 or 500 MHz or at 100 or 125 MHz, respectively) and are reported relative to deuterated solvent signals. Data for ^{1}H NMR spectra are reported as follows: chemical shift (δ ppm), multiplicity, coupling constant (Hz), and integration. Data for ^{13}C NMR spectra are reported in terms of chemical shift. IR spectra were recorded on a Varian 640-IR spectrometer and are reported in terms of frequency of absorption (cm^{-1}). Irradiations were performed with a 690 nm LED illuminator, mounted on a heatsink with lens (set is sold together as L690-66-60-550, Marubeni, Inc.) and powered by an EPS-600 Mini-Power Supply (C.B.S. Scientific Company, Inc.). Light intensity measurements were performed with a Thorlabs PM200 optical power and energy meter fitted with a S120VC standard Si photodiode power sensor (200-1100 nm, 50 mW). For O_2 concentration dependent release studies, a Piccolo2 (Pyro Science GmbH) fiber-optic oxygen meter was used to measure solution oxygen levels. Absorbance traces were taken on a Shimadzu UV-2550 spectrophotometer operated by UVProbe 2.32 software. Fluorescence traces were recorded on a PTI QuantaMaster steady-state spectrofluorometer operated by FelixGX 4.0.3 software with 2 nm excitation and emission slit widths and a 0.1 s integration rate. 96-well plate-based absorbance and fluorescence measurements were taken on a BioTek SynergyMx multi-mode microplate reader operated by Gen5 2.01 software using Corning® 96-well black, clear bottom polystyrene plates. HPLC analyses were performed on an Agilent 1260 Infinity HPLC utilizing a Gemini 5 μM C6 Phenyl 110 Å (4.6 × 250 mm) column (Phenomenex, Inc.) with a gradient of 5→95% (5.5 min) to 95→5% (1.5 min) MeCN/0.1% aqueous formic acid at a flow rate of 2.0 mL/min. Data analysis and curve fitting were performed using MS Excel 2011 and GraphPad Prism 6. Where errors are reported, chemical experiments were performed in triplicate (n = 3) and biological experiments in quadruplicate (n = 4). Mean values are reported ± s.d. Error bars in plots represent s.d. Statistical differences (p values) were derived from 2-factor ANOVA analysis using GraphPad Prism 6.

Synthetic Procedures

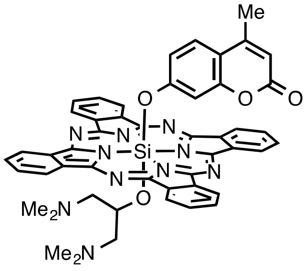

$SiPc[C_3H_5(NMe_2)_2O](C_{10}H_7O_3)$. 4-methylumbelliferone (435 mg, 2.47 mmol) was added to a solution of SiPc **1**[11] (518 mg, 0.62 mmol) in $CHCl_3$ (45 mL, neutralized over K_2CO_3). The reaction mixture was equally divided between four 20 mL vials, capped, and then stirred at 85 °C for 18 h. After 18 h, the reaction mixtures were combined, diluted with CH_2Cl_2 (50 mL), washed with saturated aqueous $NaHCO_3$ (20 mL), dried (Na_2SO_4), and concentrated on a rotary evaporator. The crude compound was purified by flash chromatography (basic alumina, 0→10% 0.1% Et_3N in CH_2Cl_2/EtOAc) to afford **$SiPc[C_3H_5(NMe_2)_2O](C_{10}H_7O_3)$** (436 mg, 81%) as a blue-green solid: ^1H NMR ($CDCl_3$, 400 MHz) δ 9.53 (dd, *J* = 5.8, 3.0 Hz, 8H), 8.25−8.18 (m, 8H), 5.69 (d, *J* = 8.7 Hz, 1H), 5.41 (s, 1H), 2.41 (d, *J* = 2.2 Hz, 1H), 2.27 (dd, *J* = 8.7, 2.3 Hz, 1H), 1.55 (s, 3H), 0.40 (s, 12H), -0.77 (dd, *J* = 12.6, 5.1 Hz, 2H), -1.59 (dd, *J* = 12.8, 5.5 Hz, 2H), -2.61− -2.68 (m, 1H); ^{13}C NMR ($CDCl_3$, 100 MHz) δ 161.2, 154.4, 152.9, 152.0, 149.7, 135.9, 131.0, 123.7, 122.8, 114.9, 111.9, 110.8, 105.4, 62.1, 61.9, 45.0, 18.0; IR ν_{max} 699, 733, 1066, 1078, 1123, 1278, 1290, 1335, 1604, 1712 cm^{-1}; HRMS (ESI) *m/z* $[M + H]^+$ 861.3083 ($C_{49}H_{40}N_{10}O_4Si + H^+$ requires 861.3076).

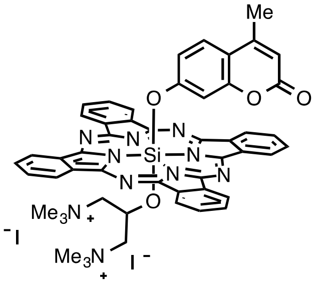

{**$SiPc[C_3H_5(NMe_3)_2O](C_{10}H_7O_3)\}I_2$ (2)**. Methyl iodide (28 mL, 890 mmol) was added to a stirring solution of **$SiPc[C_3H_5(NMe_2)_2O](C_{10}H_7O_3)$** (435 mg, 0.51 mmol) in $CHCl_3$ (56 mL, neutralized over K_2CO_3). The reaction mixture was divided equally between five 20 mL vials, sealed, and then stirred at 85 °C for 6 h. After 6 h, the reaction mixtures were allowed to cool to rt, combined, concentrated on a rotary evaporator, and then recrystallized (1:4 CH_2Cl_2/hexanes) to provide **2** (458 mg, 79%) as a blue-green solid: ^1H NMR (DMSO-d_6, 500 MHz) δ 9.79 (dd, *J* = 5.6, 3.0 Hz, 8H), 8.65 (dd, *J* = 5.7, 2.9 Hz, 8H), 6.01 (d, *J* = 8.7 Hz, 1H), 5.66 (d, *J* = 1.6 Hz, 1H), 2.25 (dd, *J* = 8.7, 2.3 Hz, 1H), 2.10 (d, *J* = 2.3 Hz, 1H), 1.69 (d, *J* = 1.3 Hz, 3H), 1.25 (s, 18H), 0.82 (dd, *J* = 13.9, 5.9 Hz, 2H), -0.35−-0.41 (m, 1H), -0.92 (dd, *J* = 13.9, 5.1 Hz, 2H); ^{13}C NMR (DMSO-d_6, 125 MHz) δ 159.1, 152.32, 152.27, 152.1, 149.3, 134.4, 133.1, 124.2, 124.0, 113.8, 111.9, 110.7, 103.9, 64.5, 57.7, 51.9, 17.4; IR ν_{max} 647, 663, 736, 979, 1059, 1121, 1289, 1335, 1600, 1709 cm^{-1}; HRMS (ESI) *m/z* $[M^{+2}]$ 445.1731 ($C_{51}H_{46}N_{10}O_4Si^{+2}$ requires 445.1731).

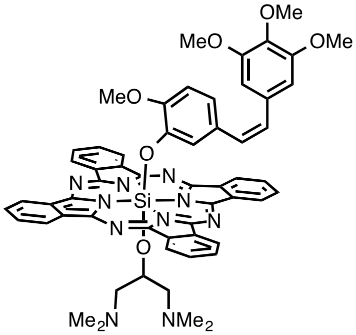

$SiPc[C_3H_5(NMe_2)_2O](C_{18}H_{19}O_5)$. *Z*-combretastatin A4 (70 mg, 0.22 mmol) was added to a stirring solution of SiPc **1**[11] (100 mg, 0.12 mmol) in $CHCl_3$ (6 mL, neutralized over K_2CO_3). The reaction mixture was stirred at 95 °C for 40 h, diluted with CH_2Cl_2 (25 mL), washed with saturated aqueous $NaHCO_3$ (10 mL), dried (Na_2SO_4), and concentrated on a rotary evaporator. The crude compound was purified by flash chromatography (basic alumina, 0→10% 0.1% Et_3N in CH_2Cl_2/EtOAc) to afford **$SiPc[C_3H_5(NMe_2)_2O](C_{18}H_{19}O_5)$** (97.7 mg, 81%) as a blue-green solid: ^1H NMR ($CDCl_3$, 400 MHz) δ 9.60 (dd, *J* = 5.6, 3.0 Hz, 8H), 8.30 (dd, *J* = 5.7, 2.9 Hz, 8H), 5.85 (s, 2H), 5.74−5.57 (m, 2H), 4.98 (dd, *J* = 24.8, 10.3 Hz, 2H), 3.73 (s, 3H), 3.31 (s, 6H), 2.16 (s, 4H), 0.49 (s, 12H), -0.73 (dd, *J* = 12.6, 5.3 Hz, 2H), -1.35−-1.63 (m, 2H), -2.59 (q, *J* = 5.3 Hz, 1H); ^{13}C NMR ($CDCl_3$, 100 MHz) δ 152.2, 149.7, 147.5, 138.2, 136.6, 136.0, 132.3, 130.6, 128.6, 127.3, 126.6, 123.4, 120.0, 119.7, 107.7, 105.5, 61.9, 61.6, 60.8, 55.6, 53.2, 45.0; IR ν_{max} 738, 1077, 1121, 1290, 1334, 1428, 1505 cm^{-1}; HRMS (ESI) *m/z* $[M + H]^+$ 1001.3899 ($C_{57}H_{52}N_{10}O_6Si + H^+$ requires 1001.3913).

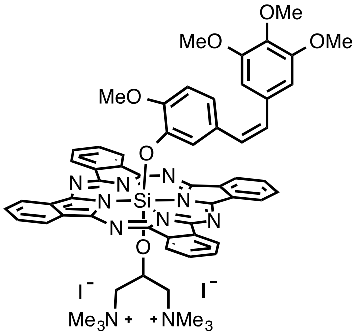

{**$SiPc[C_3H_5(NMe_3)_2O](C_{18}H_{19}O_5)\}I_2$ (3)**. Methyl iodide (5 mL, 80 mmol) was added to a stirring solution of **$SiPc[C_3H_5(NMe_2)_2O](C_{18}H_{19}O_5)$** (97.7 mg, 0.098 mmol) in $CHCl_3$ (9 mL, neutralized over K_2CO_3). The reaction mixture was stirred at 85 °C for 2 h, concentrated on a rotary evaporator, and then recrystallized (1:4 CH_2Cl_2/hexanes) to provide **3** (94.6 mg, 75%) as a blue-green solid: ^1H NMR (DMSO-d_6, 500 MHz) δ 9.71 (dd, *J* = 5.7, 3.0 Hz, 8H), 8.60 (dd, *J* = 5.8, 2.9 Hz, 8H), 5.76 (s, 2H), 5.68–5.55 (m, 2H), 5.13 (d, *J* = 8.3 Hz, 1H), 4.96 (d, *J* = 12.3 Hz, 1H), 3.64 (s, 3H), 3.15 (s, 6H), 2.07 (s, 3H), 2.03 (d, *J* = 2.2 Hz, 1H), 1.33 (s, 18H), 0.77 (dd, *J* = 13.8, 5.8 Hz, 2H), -0.51 (p, *J* = 5.6 Hz, 1H), -0.94 (dd, *J* = 13.8, 5.3 Hz, 2H); ^{13}C NMR (DMSO-d_6, 125 MHz) δ 152.6, 149.6, 147.0, 136.97, 136.92, 135.0, 133.2, 131.5, 127.51, 127.49, 127.47, 124.4, 121.3, 118.3, 109.0 ,105.8, 64.9, 60.5, 58.2, 55.7, 53.7, 52.4; IR ν_{max} 676, 736, 1122, 1292, 1335, 1429, 1504 cm^{-1}; HRMS (ESI) *m/z* $[M^{2+}]$ 515.2150 ($C_{59}H_{58}N_{10}O_6Si^{2+}$ requires 515.2148).

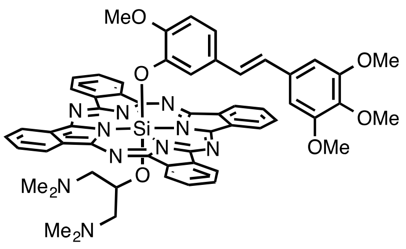

SiPc[$C_3H_5(NMe_2)_2O$](E-CA4). *E*-Combretastatin A4[12] (42 mg, 0.13 mmol) was added to a solution of SiPc **1**[11] (49.5, 0.060 mmol) in $CHCl_3$ (3 mL, neutralized over K_2CO_3). The reaction mixture was stirred at 95 °C for 40 h, diluted with CH_2Cl_2 (5mL), washed with saturated aqueous $NaHCO_3$ (2 mL), dried (Na_2SO_4), and concentrated on a rotary evaporator. The crude compound was purified by flash chromatography (basic alumina, 0→10% 0.1% Et_3N in CH_2Cl_2/EtOAc) to afford **SiPc[$C_3H_5(NMe_2)_2O$]($C_{18}H_{19}O_5$)** (46.4 mg, 78%) as a blue-green solid: ^{1}H NMR ($CDCl_3$, 400 MHz) δ 9.54 (dt, *J* = 5.8, 2.8 Hz, 8H), 8.19 (dt, *J* = 5.8, 2.8 Hz, 8H), 6.23 (s, 2H), 5.70 (d, *J* = 16.1 Hz, 1H), 5.62 (d, *J* = 7.9 Hz, 1H), 5.34 (d, *J* = 16.2 Hz, 1H), 5.00 (d, *J* = 6.9 Hz, 1H), 3.87 (s, 6H), 3.80 (s, 3H), 2.37 (s, 1H), 2.16 (s, 3H), 0.42 (s, 12H), -0.78 (ddd, *J* = 13.1, 5.2, 2.4 Hz, 2H), -1.49–-1.72 (m, 2H), -2.57–-2.76 (m, 1H); ^{13}C NMR ($CDCl_3$, 100 MHz) δ 153.1, 149.8, 148.5, 139.0, 137.2, 136.1, 133.7, 130.7, 127.8, 127.3, 124.4, 123.5, 119.5, 115.2, 108.2, 103.1, 62.0, 61.7, 61.1, 56.2, 53.4, 45.1; IR ν_{max} 731, 759, 953, 1075, 1120, 1288, 1334, 1427, 1504 cm^{-1}; HRMS (ESI) *m/z* [M + H]$^+$ 1001.3929 ($C_{57}H_{52}N_{10}O_6Si$ + H^+ requires 1001.3913).

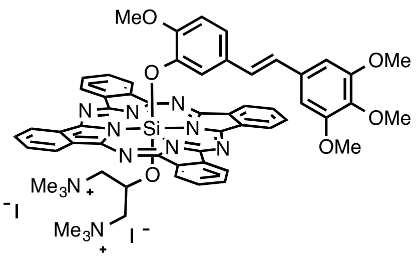

{**SiPc[$C_3H_5(NMe_3)_2O$](E-CA4)}I_2 (4)**. Methyl iodide (1.3 mL, 21.1 mmol) was added to a stirring solution of **SiPc[$C_3H_5(NMe_2)_2O$](E-CA4)** (24 mg, 0.024 mmol) in $CHCl_3$ (2.6 mL, neutralized over K_2CO_3). The reaction mixture was stirred at 85 °C for 2 h, concentrated on a rotary evaporator, and then recrystallized (1:4 CH_2Cl_2/hexanes) to provide **4** (24.4 mg, 79%) as a blue-green solid: ^{1}H NMR (DMSO-d_6, 500 MHz) δ 9.76 (dd, *J* = 5.7, 3.0 Hz, 8H), 8.60 (dd, *J* = 5.8, 2.9 Hz, 8H), 6.36 (s, 2H), 5.94 (d, *J* = 16.3 Hz, 1H), 5.81 (dd, *J* = 8.4, 2.1 Hz, 1H), 5.28–5.14 (m, 2H), 3.92 (s, 6H), 3.70 (s, 3H), 2.20 (d, *J* = 2.2 Hz, 1H), 2.18 (s, 3H), 1.38 (s, 18H), 0.83 (dd, *J* = 13.8, 5.8 Hz, 2H), -0.43 (t, *J* = 5.5 Hz, 1H), -0.88 (dd, *J* = 13.8, 5.2 Hz, 2H); ^{13}C NMR (DMSO-d_6, 125 MHz) δ 153.3, 149.9, 147.8, 137.8, 137.3, 135.0, 133.3, 133.0, 128.1, 127.1, 124.6, 124.5, 120.9, 113.8, 109.4, 103.6, 65.0, 60.6, 58.2, 56.5, 53.8, 52.4; IR ν_{max} 735, 759, 1007, 1023, 1121, 1334, 1428, 1505, 1578 cm^{-1}; HRMS (ESI) *m/z* [M^{2+}] 515.2153 ($C_{59}H_{58}N_{10}O_6Si^{2+}$ requires 515.2148).

Supplementary References

1. Matsui, T., Kitagawa, Y., Okumura, M. & Shigeta Y. Accurate standard hydrogen electrode potential and applications to the redox potentials of vitamin C and NAD/NADH. *J. Phys. Chem. A* **119**, 369-376 (2015).

2. Millis, K. K., Weaver, K. H. & Rabenstein, D. L. *J. Org. Chem.* **58**, 4144-4146 (1993).

3. Jocelyn, P. C. The standard redox potential of cysteine-cystine from the thiol-disulphide exchange reaction with glutathione and lipoic acid. *Eur. J. Biochem.* **2**, 327-331 (1967).

4. Kutnetsova, N. A., *et al.* New reagents for determination of the quantum efficiency of singlet oxygen generation in aqueous media. *Russ. J. Gen. Chem.* **71**, 36-41 (1999).

5. Davila, J. & Harriman, A. Photosensitized oxidation of biomaterials and related model compounds. *Photochem. Photobiol.* **50**, 29-35 (1989).

6. He, J. *et al.* A genetically targetable near-infrared photosensitizer. *Nat Methods*, **50**, 263-268.

7. Masilela, N., Idowu, M. & Nyokong, T. Photophysical, photochemical, and electrochemical properties of water soluble silicon, titanium and zinc phthalocyanines. *J. Photochem. Photobiol. A: Chem.* **201**, *91*-97 (2009).

8. da Silva, E. F., *et al.* Intracellular singlet oxygen photosensitizers: on the road to solving the problems of sensitizer degradation, bleaching and relocalization. *Integr. Biol.* **8**, 177-193 (2016).

9. Aveline, B., Hasan, T. & Redmond, R.W. Photophysical and photosensitizing properties of benzoporphyrin derivative monoacid ring A (BPD-MA). *Photochem. Photobiol.* **59**, 328-335 (1994).

10. Peng, X., *et al* Phthalocyanine dye as an extremely photostable and highly fluorescent near-infared labling reagent *Proceedings of SPIE*, **6097**, 6097OE-1 (2006).

11. Lo, P. C., *et al.* New amphiphilic silicon(IV) phthalocyanines as efficient photosensitizers for photodynamic therapy: synthesis, photophysical properties, and in vitro photodynamic activities. *Chem. Eur. J.* **10**, 4831-4838 (2004).

12. Gaukroger, K., Hadfield, J. A., Hepworth, L. A.., Lawrence, N. J. & McGown, A. T. Novel syntheses of cis and trans isomers of combretastatin A-4. *J. Org. Chem.* **66**, 8135-8138 (2001).
